# A Convenient One‐Pot CuI‐Catalyzed Procedure for the Synthesis of *S*‐(*N*‐Heteroaryl) Thiocarboxylates From *N*‐Heteroaryl Bromides and Thiobenzoic Acid

**DOI:** 10.1002/open.70186

**Published:** 2026-03-29

**Authors:** Jiamin Pan, Suting Xie, Yong Li, Jiang Wu, Guilong Zhao

**Affiliations:** ^1^ School of Pharmaceutical Sciences Southern Medical University Guangzhou Guangdong Province China; ^2^ Zhongshan Institute for Drug Discovery Shanghai Institute of Materia Medica Chinese Academy of Sciences Zhongshan Guangdong Province China; ^3^ College of Pharmacy Shenzhen Technology University Shenzhen Guangdong Province China; ^4^ Shanghai Institute of Materia Medica Chinese Academy of Sciences Shanghai China

**Keywords:** cross‐coupling, cuI catalysis, one‐pot procedure, *s*‐aryl thiocarboxylate, synthetic methods

## Abstract

Aryl thiols constitute a crucial class of structural motifs and synthetic building blocks, while *S*‐aryl thiocarboxylates represent an important class of latent aryl thiol precursors. CuI‐catalyzed Ullmann‐type cross coupling of aryl halides and thiobenzoic acid is a widely used synthetic method for *S*‐aryl thiocarboxylates. Inspired by two individual methods reported earlier and associated drawbacks, a convenient one‐pot two‐step CuI‐catalyzed procedure for the synthesis of *S*‐(*N*‐heteroaryl) thiocarboxylates from *N*‐heteroaryl bromides and thiobenzoic acid was developed by combining of the two reaction systems and subsequent systematic optimization of the reaction conditions. This procedure employed CuI as a transition metal catalyst, a substoichiometric amount of NaI as iodide source and *N*,*N’*‐dimethyl‐1,2‐ethanediamine and 1,10‐phenanthroline as two ligands sequentially in each step. This one‐pot procedure is highlighted by shorter reaction time, less iodide source and scalability compared to the two reported procedures while maintaining comparable overall isolated yields. Its utility was demonstrated by providing an alternative synthetic route to **25** and **26**, two dual serotonin/dopamine receptor modulators, with fewer reaction steps and avoidance of harsh reaction conditions.

## Graphical abstract

A one‐pot two‐step CuI‐catalyzed procedure for the synthesis of *S*‐(*N*‐heteroaryl) thiocarboxylates from *N*‐heteroaryl bromides and thiobenzoic acid was developed, which is highlighted by shorter reaction time, a smaller amount of iodide source and scalability.

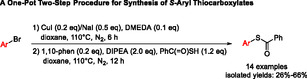

## 1 Introduction

**FIGURE 1:**
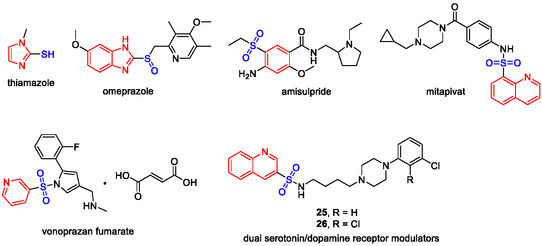
Selected drugs and biologically active compounds bearing arylthiol, arylsulfoxide and arylsulfonyl motifs.

Aryl thiols and derivatives thereof, such as arylsulfoxide or arylsulfonyl compounds, constitute an important class of structural motifs and synthetic building blocks in sulfur‐containing natural products and biologically active compounds and attract increasing attention. Some drugs and biologically active compounds with aryl thiol, arylsulfoxide, and arylsulfonyl motifs are shown in Figure 1. Traditional methods for the synthesis of aryl thiols include conversion of phenols to corresponding thiophenols by thermal rearrangement of dialkylthiocarbamates and thiocarbonates, which, however, suffers from extreme reaction conditions (up to 335°C) and thus limited substrate scope [1, 2, 3]. Transition metal‐catalyzed Ullmann‐type Ar—S bond formation by cross coupling reaction of aryl halides and alkyl thiols emerged in the 1980s [4], and the products alkyl aryl thioethers were subsequently cleaved to give desired aryl thiols under harsh conditions, such as use of reactive metals (Na [5, 6] and Mg [7]) or excess alkyl thiols at high temperatures (refluxing DMF) [5], largely limiting their wide applicability.

**SCHEME 1:**
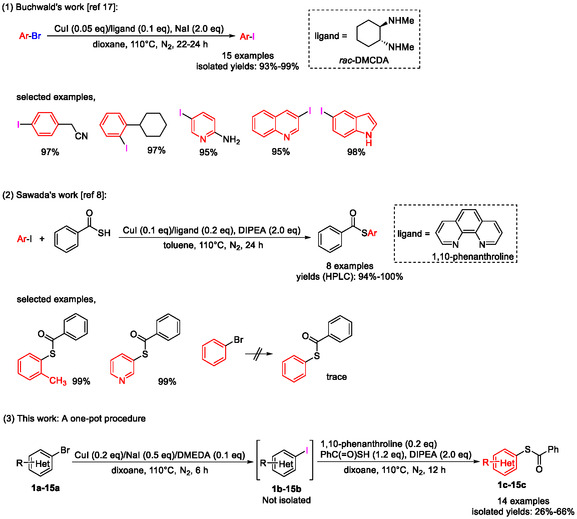
Synthesis of *S*‐aryl thiocarboxylates from aryl bromides.

In light of this, efficient methods for the synthesis of *S*‐aryl thiocarboxylates are highly desirable because they can be readily hydrolyzed to aryl thiols under mild alkaline conditions [8], although many other sulfur sources as well as related thiol liberation strategies have been reported in the transition metal‐catalyzed synthesis of aryl thiols, such as thiourea [9], 2‐(pyridin‐3‐yl)ethane‐1‐thiol [10], *β*‐mecaptopropionates [10], triisopropylsilylthiol (TIPS‐SH) [11], Na_2_S_2_O_3_ [12], Na_2_S [13], sulfur powder [14], 1,2‐ethanedithiol [15], and H_2_S [16]. Among the reported methods for the synthesis of *S*‐aryl thiocarboxylates, CuI‐catalyzed Ullmann‐type cross coupling of aryl halides and thiobenzoic acid represents an important method due to the mild reaction conditions and wide substrate scope. This method suffers from one major drawback: only aryl iodides, not aryl bromides, could be used as substrates since the latter were not reactive enough (Scheme 1, part (2)) [8]; however, aryl iodides are usually much less commercially available and significantly more expensive than corresponding bromides, necessitating that the synthesis of *S*‐aryl thiocarboxylates from aryl bromides needs two separate steps, i.e., initial conversion of aryl bromides to corresponding aryl iodides, followed by their conversion to *S*‐aryl thiocarboxylates. Fortunately, convenient conversion of aryl bromides to aryl iodides catalyzed by CuI has been reported (Scheme 1, part (1)) [17]. Inspired by the observation that both conversions shared identical transition metal catalyst (CuI) and reaction temperature (110°C), and similar bidentate ligands (*rac*‐DMCDA and 1,10‐phenanthroline), we envisioned that these two conversions might be achieved in a one‐pot manner. Thus, after systematic screenings of reaction conditions and substrate scope, we successfully developed a convenient one‐pot two‐step procedure for the synthesis of *S*‐(*N*‐heteroaryl) thiocarboxylates from *N*‐heteroaryl bromides (Scheme 1, part (3)), which has been validated on a multi‐gram scale and demonstrated its utility in the synthesis of two dual serotonin/dopamine receptor modulators **25** and **26** with significant advantages (Figure 1). This straightforward one‐pot procedure was highlighted by shorter reaction time, reduced iodide consumption and scalability compared to the reported two separate reactions while maintaining comparable overall yields.

## 2 Results and Discussion

The development of the present one‐pot synthetic procedure for *S*‐aryl thiocarboxylates from aryl bromides and thiobenzoic acid was based on two previously reported individual procedures (Scheme 1, part (1) and (2)). Thus, the conversion of aryl bromides to corresponding aryl iodides was achieved by reacting aryl bromides (1.0 eq) with NaI (2.0 eq, iodide source) in the presence of CuI (0.05 eq, copper catalyst) and *rac*‐DMCDA (racemic *N1*,*N2*‐dimethylcyclohexane‐1,2‐diamine, 0.1 eq, ligand 1) in dioxane at 110°C for 22–24 h (Scheme 1, part (1)) [17], while the conversion of aryl iodides to corresponding *S*‐aryl thiocarboxylates was achieved by treatment of aryl iodides (1.0 eq) with thiobenzoic acid (1.2 eq, sulfur source) and diisopropylethylamine (DIPEA, 2.0 eq, base) in the presence of CuI (0.1 eq, copper catalyst) and 1,10‐phenanthroline (0.2 eq, ligand 2) in toluene at 110°C for 24 h (Scheme 1, part (2)) [8]. The drawbacks associated with these two procedures are long reaction time (46–48 h in total), complex operations involving workup and purification of the aryl iodide intermediates by column chromatography and excess costly iodide source (NaI, 2.0 eq).

Inspired by the observation that, despite different reaction mechanisms, both steps shared the same transition metal catalyst (CuI), similar bidentate ligands (*rac*‐DMCDA and 1,10‐phenanthroline) and same reaction temperature (110°C), we envisioned that these two conversions might be achieved in a one‐pot manner after careful optimizations. The optimization process using 3‐bromopyridine (**1a**) as starting aryl bromide template was summarized in Table 1. Thus, **1a** (1.0 eq) was treated with thiobenzoic acid (1.2 eq) and DIPEA (2.0 eq) in the presence of CuI (0.1 eq) and 1,10‐phenanthroline (0.2 eq) in toluene at 110°C for 24 h to find that only trace amount of desired **1c** was formed (Entry 1, Table 1), confirming the earlier observation that aryl bromides were not reactive enough for CuI‐catalyzed coupling with thiobenzoic acid [8]. For the development of the envisioned one‐pot procedure, direct combination of two reaction systems into a one‐pot one‐step procedure was initially attempted given their similarity discussed above (Entry 2–3, Table 1). Thus, a mixture of **1a** (1.0 eq), thiobenzoic acid (1.2 eq), DIPEA (2.0 eq), CuI (0.2 eq), NaI (2.0 eq), DMCDA (0.1 eq) or *N*,*N’*‐dimethyl‐1,2‐ethanediamine (DMEDA, 0.1 eq), and 1,10‐phenanthroline (0.2 eq) in dioxane was stirred at 110°C for 24 h, but it turned out that no desired **1c** was formed because the reaction mixture solidified during the reaction leading to hard stirring, which suggested that direct combination of the two reaction systems into a one‐pot one‐step procedure was infeasible since they were not compatible. We then turned to the development of a one‐pot two‐step procedure, where step 2 was initiated only after step 1 finished but no additional CuI was added for step 2 because the CuI for step 1 could be further used in step 2. Thus, a mixture of **1a** (1.0 eq), CuI (0.2 eq), NaI (2.0 eq) and DMEDA (0.1 eq) in dioxane was stirred at 110°C for 6 h, when **1a** was completely consumed, and thiobenzoic acid (1.2 eq), DIPEA (2.0 eq), and ligand 2 (0.2 eq) were subsequently added to the reaction mixture to initiate step 2 at the same temperature, which typically finished within 12 h as indicated by TLC analysis (Entry 4, Table 1). Surprisingly, this one‐pot two‐step proceeded well with an isolated yield of 44%. The equivalents of NaI for step 1 were then optimized because it was expensive and initially used in more than stoichiometric amount (Entry 5–10, Table 1), and it was found that the isolated yield kept steady (41%‐48%) when NaI ranged from 0.5 eq to 1.5 eq, while 0.25 eq and 3.0 eq were associated with significant drop of isolated yield. Therefore, 0.5 eq was selected as the optimal equivalent for NaI. It should be noted that theoretically at least 1.0 eq of NaI is necessary for step 1 to complete, and the observation that the isolated yield for 0.5 eq of NaI was almost the same as those for 1.0 eq and 1.5 eq may be explained by the possibility that NaI re‐generated in step 2 could be recycled for step 1. Actually, it was experimentally found that when 0.5 eq of NaI was used for step 1 (Entry 6, Table 1), the conversion of **1a** to **1b** was about 50% (**1a**/**1b** = 45.48%/54.52%) as indicated by liquid chromatography‐mass spectrometry (LC‐MS), but all **1a** was consumed when step 2 was finished. This is one major advantage in the present one‐pot two‐step procedure compared with two separate procedures since NaI is expensive. When the equivalent of CuI was decreased from 0.2 eq (Entry 6, Table 1) to 0.1 eq (Entry 11, Table 1), the isolated yield decreased from 43% to 34%, indicating that 0.2 eq of CuI was necessary to maintain this one‐pot procedure efficient. An attempt to combine the conditions of both steps in Entry 6 into a one‐pot one‐step procedure described above as in Entry 2 and 3 failed once again in that no desired **1c** was observed and the reaction mixture also solidified, leading to difficult stirring, which further indicated that the two reaction systems were not compatible (Entry 12, Table 1). Switch of ligand 1, DMEDA, to 1,3‐propanediamine (1,3‐PDA), another efficient ligand earlier reported for step 1 [17], led to dramatic drop in isolated yield (Entry 13, Table 1). Attempts to use one single ligand in both steps were also unsuccessful, indicating that, although both DMEDA and 1,10‐phenanthroline belong to N‐based bidentate ligands, their exact functionalities differed to a large extent for their own steps and a two‐ligand system was necessary (Entry 14 and 15, Table 1). Considering the reported solvent for step 2 was toluene [8], we screened the solvent for this step using a mixed dioxane/toluene solvent system with varied ratios as well as DMF (Entry 16–20, Table 1), and it turned out that the isolated yield decreased as the ratio of toluene increased in mixed dioxane/toluene solvent system, and when DMF was used as solvent, no desired **1c** was observed. Finally, a variety of bases for step 2 were screened to find out that K_3_PO_4_ was associated with a comparable yield to DIPEA, while Et_3_N led to significant decrease of isolated yield and Cs_2_CO_3_ and *t*‐BuONa led to complete loss of isolated yield (Entry 21–24, Table 1). Noteworthy is that when Cs_2_CO_3_ and *t*‐BuONa were used as base for step 2, like in the one‐pot one‐step procedures discussed above (Entry 2, 3, and 12), difficult stirring of reaction mixture also occurred. In summary, the optimized reaction conditions for the present one‐pot two‐step procedure was: A mixture of **1a** (1.0 eq), CuI (0.2 eq), NaI (0.5 eq) and DMEDA (0.1 eq) in dioxane was stirred at 110°C for 6 h, when most of the **1a** was consumed and the conversion of **1a** to **1b** plateaued as indicated by LC‐MS, and thiobenzoic acid (1.2 eq), DIPEA (2.0 eq) and 1,10‐phenanthroline (0.2 eq) were subsequently added to the reaction mixture to initiate step 2 at the same temperature, which typically finished within 12 h as indicated by TLC analysis (Entry 6, Table 1).

**TABLE 1:** Screening of reaction conditions for CuI‐catalyzed cross coupling of aryl bromides and thiobenzoic acid.

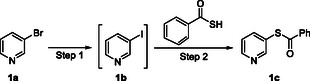							
Entry	Condition for Step 1^a^				Condition for Step 2^b^		Yield^c^, %
	CuI, eq	NaI, eq	Ligand 1, (0.1 eq)	Solvent	Ligand 2 (0.2 eq)	Base (2.0 eq)	
1^d^	0.1	—	—	PhCH_3_	1,10‐phen^e^	DIPEA^f^	Trace
2^g^	0.2	2.0	DMCDA^h^	Dioxane	1,10‐phen	DIPEA	0^i^
3^g^	0.2	2.0	DMEDA^j^	Dioxane	1,10‐phen	DIPEA	0^i^
4	0.2	2.0	DMEDA	Dioxane	1,10‐phen	DIPEA	44
5	0.2	0.25	DMEDA	Dioxane	1,10‐phen	DIPEA	35
6	0.2	0.5	DMEDA	Dioxane	1,10‐phen	DIPEA	43
7	0.2	0.75	DMEDA	Dioxane	1,10‐phen	DIPEA	41
8	0.2	1.0	DMEDA	Dioxane	1,10‐phen	DIPEA	44
9	0.2	1.5	DMEDA	Dioxane	1,10‐phen	DIPEA	48
10	0.2	3.0	DMEDA	Dioxane	1,10‐phen	DIPEA	34
11	0.1	0.5	DMEDA	Dioxane	1,10‐phen	DIPEA	34
12^g^	0.2	0.5	DMEDA	Dioxane	1,10‐phen	DIPEA	0^i^
13	0.2	0.5	1,3‐PDA^k^	Dioxane	1,10‐phen	DIPEA	17
14	0.2	0.5	1,10‐phen	Dioxane	—	DIPEA	6
15	0.2	0.5	DMEDA	Dioxane	—	DIPEA	3
16	0.2	0.5	DMEDA	Dioxane/ PhCH_3_ (9/1)	1,10‐phen	DIPEA	42
17	0.2	0.5	DMEDA	Dioxane/ PhCH_3_ (4/1)	1,10‐phen	DIPEA	33
18	0.2	0.5	DMEDA	Dioxane/ PhCH_3_ (1/1)	1,10‐phen	DIPEA	13
19	0.2	0.5	DMEDA	PhCH_3_	1,10‐phen	DIPEA	6
20	0.2	0.5	DMEDA	DMF	1,10‐phen	DIPEA	0
21	0.2	0.5	DMEDA	Dioxane	1,10‐phen	Cs_2_CO_3_	0^i^
22	0.2	0.5	DMEDA	Dioxane	1,10‐phen	*t*‐BuONa	0^i^
23	0.2	0.5	DMEDA	dioxane	1,10‐phen	K_3_PO_4_	40
24	0.2	0.5	DMEDA	Dioxane	1,10‐phen	Et_3_N	33

a Step 1: A mixture of **1a** (1.000 g, 1.0 eq), CuI, NaI, and ligand 1 (0.1 eq) in solvent (20 mL) was stirred at 110°C under N_2_ for 6 h.

b Step 2: Thiobenzoic acid (1.2 eq), base (2.0 eq) and ligand 2 (0.2 eq) were added, and the stirring was continued at 110°C for 12 h.

c Isolated yield.

d A mixture of **1a** (1.000 g, 1.0 eq), CuI (0.121 g, 0.1 eq), 1,10‐phenanthroline (0.228 g, 0.2 eq), thiobenzoic acid (1.049 g, 1.2  eq), DIPEA (1.636 g, 2.0 eq) in toluene (20 mL) under N_2_ was stirred at 110°C for 24 h.

e 1,10‐phen = 1,10‐phenanthroline.

f DIPEA = diisopropylethylamine.

g A mixture of **1a** (1.000 g, 1 eq), CuI (0.241 g, 0.2 eq), NaI (0.5 eq or 2.0 eq), DMEDA (0.055 g, 0.1 eq) or DMCDA (0.090 g, 0.1  eq), thiobenzoic acid (1.049 g, 1.2 eq), DIPEA (1.636 g, 2.0 eq) and 1,10‐phenanthroline (0.228 g, 0.2 eq) in dioxane (25 mL) under N_2_ was stirred at 110°C for 24 h.

h DMCDA = (1*R*,2*R*)‐*N1*,*N2*‐dimethylcyclohexane‐1,2‐diamine.

i Hard stirring because the reaction mixture solidified and no desired product **1c** was observed.

j DMEDA = *N*,*N’*‐dimethyl‐1,2‐ethanediamine.

k 1,3‐PDA = 1,3‐propanediamine.

With the optimized reaction conditions in hand, we moved on to screen the substrate scope. As shown in Table 2, when the substrates were phenyl or naphthyl bromides, almost no desired *S*‐aryl thiocarboxylates were produced (**2c**–**6c**), but when the substrates were *N*‐bearing heteroaryl bromides, the desired products were produced in moderate yields (**1c**, **7c**–**19c**). These aromatic heterocycles included pyridine, quinoline, isoquinoline, quinazoline, and indole. 3‐Bromoquinoline (**8a**) was selected as substrate to explore the scalability of the present one‐pot procedure, and it was found that the yield kept steady with slight loss when the reaction was carried out on multiple‐gram scales (**8c**). The underlying reason why phenyl or naphthyl bromides failed as substrates was further studied using the conversion of 1‐bromonaphthalene (**5a**) to **5c**. Thus, the two reaction mixtures obtained after step 1 and step 2 were analyzed by LC‐MS and TLC to find that under the present one‐pot condition the conversion of 1‐bromonaphthalene (**5a**) to corresponding iodide **5b** proceeded like other substrates, but no desired product **5c** was observed in step 2, indicating that the present optimized one‐pot two‐step reaction system was not applicable to phenyl or naphthyl bromides presumably because two CuI‐ligand systems interfered with each other in such substrates, which represented a limitation of the present one‐pot procedure compared to the two separate steps. In a control experiment, commercially available iodobenzene (**2b**) was subjected to step 2 under optimized reaction condition (Entry 6, Table 1), but no desired product **2c** was observed by LC‐MS and ^1^H NMR, further supporting the explanation discussed above.

**TABLE 2:** Screening of substrate scope of CuI‐catalyzed cross coupling of aryl bromides and thiobenzoic acid.^a^

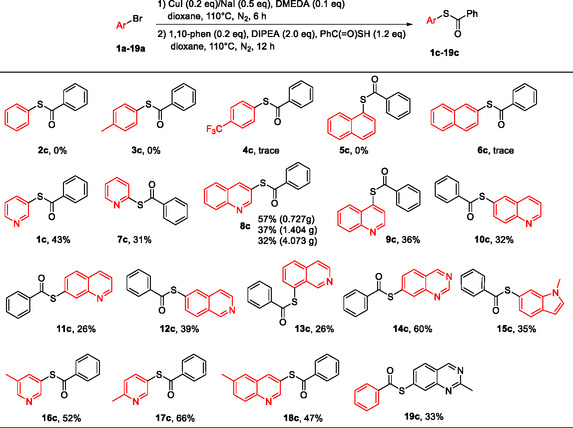	

a A mixture of aryl bromides **1a**–**19a** (1.0 eq), CuI (0.2 eq), NaI (0.5 eq) and DMEDA (0.1 eq) in dioxane (20 mL) was stirred at 110°C under N_2_ for 6 h. After that, thiobenzoic acid (1.2 eq), DIPEA (2.0 eq) and 1,10‐phenanthroline (0.2 eq, dissolved in dioxane (5 mL)) were then added successively, and the reaction mixture was stirred for additional 12 h at this temperature. All yields are isolated yields.

Given the observations that the reported yields for the two individual steps are remarkably high (>90%, isolated [17] or HPLC‐based [8]) and that the reported yields can’t usually be reproduced in reality, we moved on to carry out head‐to‐head comparison between the present one‐pot procedure and the procedure involving two separate steps (Table 3). Thus, when the selected substrates **1a**, **8a** and **14a** were subjected to the two‐pot procedure, moderate yields for step 1 and high yields for step 2 were observed, which, however, led to moderate overall yields for both steps, comparable or even lower than the yields obtained with the present one‐pot procedure. This demonstrated the advantage of the present one‐pot procedure in terms of yields although the yields seemed moderate.

**TABLE 3:** Comparison of the present one‐pot procedure and the procedure involving two separate steps.

Entry	Substrate	Product	Two‐pot procedure^a^				One‐pot procedure^b^	
			Time, h	Yield for step 1^c^	Yield for step 2^c^	Overall yield^c^	Time, h	Yield^c^
1	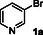	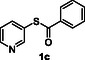	36	48%	90%	43%	18	43%
2	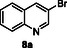	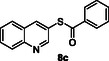	36	61%	70%	43%	18	57%
3	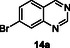	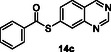	36	65%	77%	50%	18	60%

a Step 1: A mixture of aryl bromide **1a**, **8a** or **14a** (1.000 g, 1.0 eq), CuI (0.2 eq), NaI (0.5 eq), and DMEDA (0.1 eq) in dioxane (20 mL) under N_2_ was stirred at 110°C for 6 h. The reaction mixture was subjected to workup and purified by column chromatography to isolate corresponding crude aryl iodide **1b**, **8b** or **14b**, and the yield was calculated. Step 2: A mixture of crude aryl iodide **1b**, **8b** or **14b** (1.0 eq) prepared above in Step 1, CuI (0.1 eq), thiobenzoic acid (1.2 eq), DIPEA (2.0 eq) and 1,10‐phenanthroline (0.2 eq) in dioxane (20 mL) was stirred at 110°C under N_2_ for 24 h. Workup and purification by column chromatography gave *S*‐aryl thiobenzoate **1c**, **8c** or **14c**.

b A mixture of aryl bromides **1a, 8a** or **14a** (1.000 g, 1.0 eq), CuI (0.2 eq), NaI (0.5 eq), and DMEDA (0.1 eq) in dioxane (20 mL) was stirred at 110°C under N_2_ for 6 h. After that, thiobenzoic acid (1.2 eq), DIPEA (2.0 eq), and 1,10‐phenanthroline (0.2 eq, dissolved in dioxane (5 mL)) were added successively, and the reaction mixture was stirred for additional 12 h at this temperature.

c Isolated yields.

**SCHEME 2:**
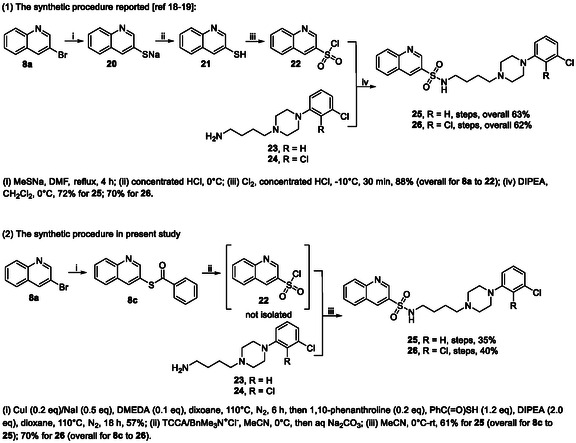
Comparison of reported and present one‐pot synthetic procedures for **25** and **26**.

Finally, we demonstrated the utility of the present one‐pot procedure by providing an alternative synthetic route to **25** and **26** (Scheme 2). Compounds **25** and **26** are dual serotonin/dopamine receptor modulators [18, 19]. Their initial reported synthetic procedures are summarized in part (1) of Scheme 2. Thus, 3‐bromoquinoline (**8a**) was converted to corresponding sodium aryl thiolate **20** by treating **8a** with a large excess amount of MeSNa in refluxing DMF, and the latter was then acidified with HCl to afford aryl thiol **21**, which was in turn oxidized to corresponding arylsulfonyl chloride **22** with Cl_2_ at −10°C in an overall yield of 88% (**8a** to **22**) [20]. DIPEA‐mediated sulfonamide formation of **22** and amine **23** and **24** gave **25** and **26**, respectively. The overall yields for **25** and **26** from **8a** are 63% and 62%, respectively. Target compounds **25** and **26** were also synthesized by a procedure based on the present one‐pot procedure (Scheme 2, part (2)). Thus, **8a** was converted to *S*‐aryl thiocarboxylate **8c** in 57% yield by the present one‐pot procedure, and the latter was oxidized to arylsulfonyl chloride **22** with trichloroisocyanuric acid (TCCA)/BnMe_3_N^+^Cl^–^ in MeCN in the presence of aqueous Na_2_CO_3_. Arylsulfonyl chloride **22** was not isolated from the reaction mixture, but was directly reacted with amine **23** and **24** to give **25** and **26** [21]. The overall yields for **25** and **26** from **8a** are 35% and 40%, respectively. In summary, although the synthetic route to **25** and **26** based on the present one‐pot procedure was associated with lower overall yields compared to the reported procedure (the reported yields were not confirmed independently in our laboratory), it was highlighted by fewer reaction steps and avoidance of harsh reaction conditions used in the reported procedure, such as high temperature (refluxing DMF) and highly toxic reagent (Cl_2_).

## 3 Conclusion

In conclusion, a convenient one‐pot CuI‐catalyzed procedure for the synthesis of *S*‐(*N*‐heteroaryl) thiocarboxylates from *N*‐heteroaryl bromides and thiobenzoic acid was developed after systematic optimization of the reaction conditions based on two separate reactions reported earlier. This procedure is highlighted by shorter reaction time, a smaller amount of iodide source and scalability compared to the reported two separate procedures while maintaining comparable overall yields. Its utility was demonstrated by providing an alterbative synthetic route to **25** and **26**, with fewer reaction steps and avoidance of harsh reaction conditions. However, this procedure has a limitation that it was not applicable to phenyl and naphthyl bromides as substrates, which is an ongoing research subject in our laboratory.

## 4 Experimental Section

### 4.1 General Methods

All the chemical reagents and organic solvents were purchased commercially and used without further purification. Reaction progress was monitored by thin‐layer chromatography (TLC) on precoated TLC silica gel plates or liquid chromatography‐mass spectrometry (LC‐MS). Purification by column chromatography was carried out on silica gel column (200–300 or 300–400 mesh) with EtOAc/petroleum ether (PE) or MeOH/CH_2_Cl_2_ (reported by v/v) as eluent system. Melting points were determined with an SGW X‐4A microscopic melting point apparatus and are uncorrected. NMR spectra were recorded on a Bruker Ascend 500 or 600 NMR spectrometer (500 MHz or 600 MHz; Bruker Switzerland AG, Fällanden, Switzerland), using CDCl_3_ or DMSO‐*d*_6_ as solvent. High‐resolution mass spectra (HR‐MS) were recorded with a Thermo Scientific Exactive Plus mass spectrometer (Thermo Fisher Scientific, Bremen, Germany) using electrospray ionization (ESI) and Orbitrap mass analyzer.

Procedure for the synthesis of 1c directly from 1a (Entry 1, Table 1)

A mixture of 3‐bromopyridine (**1a**, 1.000 g, 1.0 eq), CuI (0.121 g, 0.1 eq), 1,10‐phenanthroline (0.228 g, 0.2 eq), thiobenzoic acid (1.049 g, 1.2 eq), DIPEA (1.636 g, 2.0 eq) in toluene (20 mL) under N_2_ was stirred at 110°C for 24 h. TLC analysis indicated that only trace amount of 1c was formed. The reaction mixture was not further subjected to workup and purification process.

### 4.2 One‐Pot Two‐Step General Procedure for the Screening of Reaction Condition (Entry 4–11 and 13–24, Table 1)

Step 1: A mixture of **1a** (1.000 g, 6.33 mmol, 1.0 eq), CuI, NaI, ligand 1 (0.1 eq) in solvent (20 mL) was stirred under N_2_ at 110°C for 6 hr, when LC‐MS confirmed the reaction completed or progress stopped.
Step 2: Thiobenzoic acid (1.049 g, 7.60 mmol, 1.2 eq), base (2.0 eq) and ligand 2 (0.2 eq, dissolved in dioxane (5 mL)) were added successively at 110°C, and the reaction mixture was then stirred for 12 h at this temperature.

On cooling to room temperature, the reaction mixture was diluted with water (50 mL), and the resulting mixture was extracted with EtOAc (30 mL × 3). The combined extracts were washed with brine (35 mL), dried (Na_2_SO_4_) and evaporated on a rotary evaporator to afford a residue, which was purified by column chromatography (EtOAc/PE = 15/85 by v/v) to give **1c**.

For the reaction in entry 6, Table 1, a LC‐MS method was established to follow changes of **1a**, **1b,** and **1c** before and after the step 2. It was found that, with 0.5 eq of NaI, 54.52% of **1a** was converted to **1b** after the step 1 (before initiating step 2), and after the step 2, both **1a** and **1b** were consumed completely. The LC‐MS conditions are listed below: LC‐MS, Agilent 1260 infinity II‐IQ; Column, Gensial, C_18_, 4.6 × 50 mm, 3 μm; detector wavelength, 280 nm; flow rate, 0.4 mL/min; eluent, A = 0.1% aqueous HCO_2_H, B = MeOH, gradient elution,

**Table:** 

Time, min	A, %	B, %
0	55	45
2	55	45
12	5	95
20	5	95

### 4.3 One‐Step Procedure for the Screening of Reaction Condition (Entry 2, 3, and 12, Table 1)

A mixture of **1a** (1.000 g, 1 eq), CuI (0.241 g, 0.2 eq), NaI (0.5 eq or 2.0 eq), DMEDA (0.055 g, 0.1 eq) or DMCDA (0.090 g, 0.1 eq), thiobenzoic acid (1.049 g, 1.2 eq), DIPEA (1.636 g, 2.0 eq), and 1,10‐phenanthroline (0.228 g, 0.2 eq) in dioxane (25 mL) under N_2_ was stirred at 110°C for 24 h. On cooling to room temperature, the reaction mixture was subjected to the same workup procedure described above in the two‐step one‐pot procedure.

### 4.4 General Procedure for Substrate Scope Study under Optimized Conditions (Table 2)

A mixture of aryl bromides **1a**–**19a** (1.000 g, 1.0 eq), CuI (0.2 eq), NaI (0.5 eq), DMEDA (0.1 eq) in dioxane (20 mL) was stirred at 110°C under N_2_ for 6 h. Thiobenzoic acid (1.2 eq), DIPEA (2.0 eq) and 1,10‐phenanthroline (0.2 eq, dissolved in dioxane (5 mL)) were then added successively, and the reaction mixture was stirred for additional 12 h at this temperature, when TLC analysis indicated the completion of reaction. On cooling to room temperature, the reaction mixture was diluted with water (50 mL), and the resulting mixture was extracted with EtOAc (30 mL × 3). The combined extracts were washed with brine (35 mL), dried (Na_2_SO_4_) and evaporated on a rotary evaporator to afford a residue, which was purified by column chromatography (EtOAc/PE = 0/100 − 25/75 by v/v) to afford *S*‐aryl thiobenzoates **1c**–**19c**.

### 4.5 S‐(pyridin‐3‐yl) Benzothioate (1c)

0.588 g (43%); White Solid; m.p. 74.8°C–76.1°C.

^1^H NMR (600 MHz, DMSO‐*d*_6_) δ* =* 8.70 (d, *J* = 4.7 Hz, 1H), 8.66 (s, 1H), 8.05–7.97 (m, 3H), 7.76 (t, *J* = 5.4 Hz, 1H), 7.66–7.59 (m, 2H), 7.58–7.54 (m, 1H).

^13^C NMR (126 MHz, DMSO‐*d*_6_) δ* =* 188.41, 154.30, 150.36, 142.79, 135.41, 134.56, 129.31, 127.22, 124.46, 124.38.

HRMS (ESI): *m*/*z* calcd for C_12_H_9_NOS [M + H]^+^: 216.0478, found: 216.0477.

### 4.6 S‐(pyridin‐2‐yl) Benzothioate (7c)

0.421 g (31%); Yellow Solid; m.p. 39.8°C–41.4°C.

^1^H NMR (500 MHz, DMSO‐*d*_6_) δ* =* 8.66 (d, *J* = 4.9 Hz, 1H), 7.98 (d, *J* = 8.5 Hz, 2H), 7.96–7.92 (m, 1H), 7.78 (d, *J* = 7.9 Hz, 1H), 7.75 (d, *J* = 4.5 Hz, 1H), 7.66–7.58 (m, 2H), 7.51 (t, *J* = 6.8 Hz, 1H).

^13^C NMR (126 MHz, DMSO‐*d*_6_) δ* =* 188.72, 150.54, 150.23, 137.73, 135.85, 134.44, 130.97, 129.30, 127.10, 124.21.

HRMS (ESI): *m*/*z* calcd for C_12_H_9_NOS [M + H]^+^: 216.0478, found: 216.0477.

### 4.7 S‐(quinolin‐3‐yl) benzothioate (8c)

0.727 g (57%); White Solid; m.p. 105.1°C–106.0°C.

^1^H NMR (500 MHz, DMSO‐*d*_6_) δ *=* 8.89 (d, *J* = 2.5 Hz, 1H), 8.68 (s, 1H), 8.12 (d, *J* = 8.5 Hz, 1H), 8.08 (d, *J* = 8.5 Hz, 1H), 8.05 (d, *J* = 8.4 Hz, 2H), 7.89 (t, *J* = 7.7 Hz, 1H), 7.78 (t, *J* = 6.9 Hz, 1H), 7.73 (t, *J* = 8.1 Hz, 1H), 7.64 (t, *J* = 7.6 Hz, 2H).

^13^C NMR (126 MHz, DMSO‐*d*_6_) δ *=* 188.86, 154.14, 147.08, 142.85, 135.43, 134.63, 131.05, 129.37, 128.82, 128.20, 127.72, 127.45, 127.27, 121.08.

HRMS (ESI): *m*/*z* calcd for C_16_H_11_NOS [M+H]^+^: 266.0634, found: 266.0633.

### 4.8 S‐(quinolin‐4‐yl) benzothioate (9c)

0.460 g (36%); White Solid; m.p. 102.9°C–104.1°C.

^1^H NMR (500 MHz, DMSO‐*d*_6_) δ* =* 9.01 (d, *J* = 4.5 Hz, 1H), 8.17 (dd, *J* = 11.8, 6.3 Hz, 2H), 8.07 (dd, *J* = 8.4, 3.17 Hz, 2H), 7.90–7.83 (m, 2H), 7.83–7.76 (m, 1H), 7.70 (ddd, *J* = 8.3, 6.9, 1.3 Hz, 1H), 7.67–7.59 (m, 2H).

^13^C NMR (126 MHz, DMSO‐*d*_6_) δ* =* 187.32, 150.13, 148.17, 135.48, 135.34, 134.75, 130.20, 129.81, 129.41, 129.20, 128.60, 127.95, 127.46, 125.02.

HRMS (ESI): *m*/*z* calcd for C_16_H_11_NOS [M+H]^+^: 266.0634, found: 266.0633.

### 4.9 S‐(quinolin‐6‐yl) benzothioate (10c)

0.408 g (32%); White Solid; m.p. 143.1°C–144.2°C.

^1^H NMR (500 MHz, DMSO‐*d*_6_) δ* =* 9.01 (d, *J* = 4.3 Hz, 1H), 8.46 (d, *J* = 8.4 Hz, 1H), 8.28 (s, 1H), 8.13 (d, *J* = 8.8 Hz, 1H), 8.03 (d, *J* = 8.5 Hz, 2H), 7.83 (d, *J* = 8.8 Hz, 1H), 7.77 (t, *J* = 7.4 Hz, 1H), 7.64 (t, *J* = 7.7 Hz, 3H).

^13^C NMR (126 MHz, DMSO‐*d*_6_) δ* =* 189.11, 151.96, 147.59, 136.09, 135.72, 135.16, 135.06, 134.48, 129.89, 129.36, 128.18, 127.16, 125.03, 122.19.

HRMS (ESI): *m*/*z* calcd for C_16_H_11_NOS [M + H]^+^: 266.0634, found: 266.0633.

### 4.10 S‐(quinolin‐7‐yl) Benzothioate (11c)

0.325 g (26%); White Solid; m.p. 103.5°C–105.3°C.

^1^H NMR (500 MHz, DMSO‐*d*_6_) δ *=* 8.99 (dd, *J* = 4.5, 2.0 Hz, 1H), 8.47 (d, *J* = 8.1 Hz, 1H), 8.24 (s, 1H), 8.12 (d, *J* = 8.5 Hz, 1H), 8.03 (d, *J* = 7.1 Hz, 2H), 7.77 (t, *J* = 7.4 Hz, 1H), 7.70 (dd, *J* = 8.5, 2.0 Hz, 1H), 7.67–7.58 (m, 3H).

^13^C NMR (151 MHz, DMSO‐*d*_6_) *δ* *=* 188.85, 151.45, 147.54, 136.05, 135.74, 135.46, 134.49, 131.96, 129.35, 128.99, 128.29, 128.15, 127.19, 122.67.

HRMS (ESI): *m*/*z* calcd for C_16_H_11_NOS [M + H]^+^: 266.0634, found: 266.0640.

### 4.11 S‐(isoquinolin‐6‐yl) Benzothioate (12c)

0.498 (39%); White Solid; m.p. 140.6°C–141.2°C.

^1^H NMR (500 MHz, DMSO‐*d*_6_) δ* =* 9.05 (d, *J* = 1.9 Hz, 1H), 9.04 (d, *J* = 1.9 Hz, 1H), 8.35 (d, *J* = 2.0 Hz, 1H), 8.22 (d, *J* = 8.8 Hz, 1H), 8.05 (d, *J* = 1.3 Hz, 1H), 8.03 (d, *J* = 1.4 Hz, 1H), 7.96 (dd, *J* = 8.6, 2.0 Hz, 1H), 7.78 (t, *J* = 6.9 Hz, 1H), 7.64 (t, *J* = 7.0 Hz, 2H).

^13^C NMR (126 MHz, DMSO‐*d*_6_) δ* =* 188.49, 146.92, 146.53, 142.30, 142.06, 135.64, 135.58, 134.60, 129.92, 129.37, 127.24.

HRMS (ESI): *m*/*z* calcd for C_15_H_10_N_2_OS [M + H]^+^: 267.0587, found: 267.0584.

### 4.12 S‐(isoquinolin‐8‐yl) Benzothioate (13c)

0.333 g (26%); White Solid; m.p. 82.1°C–83.6°C.

^1^H NMR (500 MHz, DMSO‐*d*_6_) δ* =* 9.49 (s, 1H), 8.63 (d, *J* = 5.6 Hz, 1H), 8.21 (d, *J* = 8.3 Hz, 1H), 8.08 (d, *J* = 7.6 Hz, 2H), 7.97 (t, *J* = 6.5 Hz, 2H), 7.90 (t, *J* = 7.8 Hz, 1H), 7.79 (t, *J* = 7.4 Hz, 1H), 7.65 (t, *J* = 7.9 Hz, 2H).

^13^C NMR (126 MHz, DMSO‐*d*_6_) δ* =* 188.77, 149.74, 143.50, 136.86, 136.83, 136.35, 135.57, 134.50, 130.52, 130.48, 129.72, 129.29, 129.26, 128.40, 127.36, 124.92, 120.72.

HRMS (ESI): *m*/*z* calcd for C_16_H_11_NOS [M+H]^+^: 266.0634, found: 266.0630.

### 4.13 S‐(quinazolin‐7‐yl) benzothioate (14c)

0.762 g (60%); White Solid; m.p. 141.1°C–142.2°C.

^1^H NMR (500 MHz, DMSO‐*d*_6_) δ* =* 9.73 (s, 1H), 9.39 (s, 1H), 8.28 (d, *J* = 5.3 Hz, 1H), 8.04 (d, *J* = 9.6 Hz, 2H), 7.89 (d, *J* = 8.5 Hz, 1H), 7.79 (t, *J* = 6.9 Hz, 1H), 7.65 (t, *J* = 7.9 Hz, 2H).

^13^C NMR (126 MHz, DMSO‐*d*_6_) δ* =* 188.04, 160.95, 155.74, 149.09, 135.53, 134.69, 134.44, 133.81, 133.37, 129.41, 128.52, 127.28, 124.50.

HRMS (ESI): *m*/*z* calcd for C_15_H_10_N_2_OS [M + H]^+^: 267.0587, found: 267.0586.

### 4.14 S‐(1‐methyl‐1H‐indol‐6‐yl) benzothioate (15c)

0.453 g (35%); White Solid; m.p. 81.2°C–82.9°C.

^1^H NMR (500 MHz, DMSO‐*d*_6_) δ* =* 8.01 (d, *J* = 7.3 Hz, 2H), 7.72 (t, *J* = 7.3 Hz, 1H), 7.66 (s, 1H), 7.65 (d, *J* = 8.0 Hz, 1H), 7.60 (t, *J* = 7.8 Hz, 2H), 7.46 (d, *J* = 3.1 Hz, 1H), 7.13 (d, *J* = 8.1 Hz, 1H), 6.52 (d, *J* = 3.1 Hz, 1H), 3.81 (s, 3H).

^13^C NMR (126 MHz, DMSO‐*d*_6_) δ* =* 190.47, 136.61, 136.16, 134.05, 131.53, 129.21, 129.04, 126.98, 125.51, 121.02, 117.55, 117.01, 100.62, 32.53.

HRMS (ESI): *m*/*z* calcd for C_16_H_13_NOS [M + H]^+^: 268.0791, found: 268.0791.

### 4.15 S‐(5‐methylpyridin‐3‐yl) Benzothioate (16c)

0.689 g (52%); White Solid; m.p. 66.6°C–68.0°C.

^1^H NMR (500 MHz, DMSO‐*d*_6_) δ* =* 8.53 (s, 1H), 8.46 (s, 1H), 8.00 (d, *J* = 7.1 Hz, 2H), 7.82 (s, 1H), 7.79–7.72 (m, 1H), 7.67–7.57 (m, 2H), 2.36 (s, 3H).

^13^C NMR (151 MHz, DMSO‐*d*_6_) δ* =* 188.56, 151.43, 150.80, 142.86, 135.49, 134.56, 134.02, 129.34, 127.21, 123.62.

HRMS (ESI): *m*/*z* calcd for C_13_H_11_NOS [M + H]^+^: 230.0634, found: 230.0627.

### 4.16 S‐(6‐methylpyridin‐3‐yl) Benzothioate (17c)

0.873 g (66%); White Solid; m.p. 67.2°C–68.2°C.

^1^H NMR (500 MHz, DMSO‐*d*_6_) δ* =* 8.50 (s, 1H), 7.99 (d, *J* = 8.0 Hz, 2H), 7.83 (d, *J* = 8.1 Hz, 1H), 7.74 (t, *J* = 7.4 Hz, 1H), 7.60 (t, *J* = 7.8 Hz, 2H), 7.40 (d, *J* = 8.1 Hz, 1H), 2.54 (s, 3H).

^13^C NMR (151 MHz, DMSO‐*d*_6_) δ* =* 188.79, 159.43, 153.67, 142.89, 135.51, 134.50, 129.49, 127.35, 123.93, 120.89, 23.86.

HRMS (ESI): *m*/*z* calcd for C_13_H_11_NOS [M + H]^+^: 230.0634, found: 230.0627.

### 4.17 S‐(6‐methylquinolin‐3‐yl) Benzothioate (18c)

0.589 g (47%); White Solid; m.p. 101.7°C–102.6°C.

^1^H NMR (600 MHz, DMSO‐*d*_6_) δ* =* 8.89 (s, 1H), 8.61 (s, 1H), 8.03 (d, *J* = 7.9 Hz, 2H), 7.88 (d, *J* = 8.2 Hz, 1H), 7.77 (t, *J* = 7.5 Hz, 1H), 7.72 (d, *J* = 7.2 Hz, 1H), 7.63 (t, *J* = 7.8 Hz, 2H), 7.58 (t, *J* = 7.6 Hz, 1H), 2.75 (s, 3H).

^13^C NMR (151 MHz, DMSO‐*d*_6_) δ* =* 188.89, 153.13, 146.03, 143.07, 136.53, 135.46, 134.63, 130.99, 129.37, 127.76, 127.28, 127.24, 126.16, 120.88, 17.61.

HRMS (ESI): *m*/*z* calcd for C_17_H_13_NOS [M + H]^+^: 280.0791, found: 280.0782.

### 4.18 S‐(2‐methylquinazolin‐7‐yl) Benzothioate (19c)

0.421 g (33%); White Solid; m.p. 177.6°C–178.3°C.

^1^H NMR (600 MHz, DMSO‐*d*_6_) δ *=* 9.62 (s, 1H), 8.23 (d, *J* = 8.4 Hz, 1H), 8.15 (s, 1H), 8.04 (d, *J* = 8.1 Hz, 2H), 7.78 (t, *J* = 7.5 Hz, 2H), 7.65 (t, *J* = 7.8 Hz, 2H), 2.81 (s, 3H).

^13^C NMR (151 MHz, DMSO‐*d*_6_) δ *=* 188.12, 164.72, 161.00, 149.46, 135.58, 134.68, 134.09, 133.27, 132.38, 129.42, 128.38, 127.28, 122.42, 26.05.

HRMS (ESI): *m*/*z* calcd for C_16_H_12_N_2_OS [M+H]^+^: 281.0743, found: 280.0735.

### 4.19 General Procedure for the Two‐Pot Synthesis (Table 3)

Step 1: A mixture of aryl bromide **1a**, **8a,** or **14a** (1.000 g, 1.0 eq), CuI (0.2 eq), NaI (0.5 eq) and DMEDA (0.1 eq) in dioxane (20 mL) under N_2_ was stirred at 110°C for 6 h. On cooling to room temperature, the reaction mixture was poured into water (40 mL), and the resulting mixture was extracted with EtOAc (10 mL × 3). The combined extracts were washed with brine (35 mL), dried (Na_2_SO_4_) and evaporated on a rotary evaporator to afford a residue, which was purified by column chromatography (EtOAc/PE = 5/95–10/90) to give corresponding crude aryl iodide **1b**, **8b,** or **14b**. The sample was used directly in the next step without further characterization.
Step 2: A mixture of aryl iodide **1b**, **8b** or **14b** (1.0 eq) prepared above in Step 1, CuI (0.1 eq), thiobenzoic acid (1.2 eq), DIPEA (2.0 eq) and 1,10‐phenanthroline (0.2 eq) in dioxane (20 mL) was stirred at 110°C under N_2_ for 24 h, when TLC analysis indicated the completion of reaction. On cooling to room temperature, the reaction mixture was poured into water (50 mL), and the resulting mixture was extracted with EtOAc (30 mL × 3). The combined extracts were washed with brine (35 mL), dried (Na_2_SO_4_) and evaporated on a rotary evaporator to afford a residue, which was purified by column chromatography (EtOAc/PE = 10/90‐25/75) to give *S*‐aryl thiobenzoate **1c**, **8c** or **14c**.

### 4.20 S‐(pyridin‐3‐yl) Benzothioate (1c)

0.583 g (43%); White Solid; m.p. and ^1^H NMR Were Consistent with Those above

### 4.21 S‐(quinolin‐3‐yl) Benzothioate (8c)

0.545 g (43%); White Solid; m.p. and ^1^H NMR Were Consistent with Those above

### 4.22 S‐(quinazolin‐7‐yl) Benzothioate (14c)

0.640 g (50%); white solid; m.p. and ^1^H NMR were consistent with those above.

### 4.23 General Procedure for the Synthesis of Compounds 25 and 26

To a stirred mixture of BnMe_3_N^+^Cl^−^ (3.5 eq) in MeCN cooled at 0°C was added TCCA (1.2 eq), and after addition, the stirring was continued for 30 min to give a yellow solution, to which was added dropwise a solution of **8c** (1.0 eq) in MeCN, followed by addition of 1 M aqueous Na_2_CO_3_ solution (2.0 eq). The resulting mixture was stirred at this temperature for 20 min, and **23** or **24** (2.0 eq) was added. The reaction mixture was stirred for 40 min whilst warming to room temperature, when TLC analysis indicated the completion of reaction. The reaction mixture was poured into water, and the mixture thus obtained was extracted with EtOAc (10 mL × 3). The combined extracts were washed with brine, dried (Na_2_SO_4_) and evaporated on a rotary evaporator to afford a residue, which was purified by column chromatography (MeOH/CH_2_Cl_2_ = 8/92–10/90 by v/v) to give **25** or **26**.

### 4.24 N‐(4‐(4‐(3‐chlorophenyl)piperazin‐1‐yl)butyl)quinoline‐3‐sulfonamide (25)

Compound **25** was prepared from **23** (0.202 g, 0.754 mmol) and **8c** (0.100 g, 0.377 mmol) using BnMe_3_N^+^Cl^‐^ (0.245 g, 1.320 mmol), TCCA (0.105 g, 0.452 mmol) and 1 M Na_2_CO_3_ solution (0.753 mL, 0.753 mmol).

0.105 g(61%); White Solid; m.p. 116.1°C–117.8°C.

^1^H NMR (500 MHz, CDCl_3_) δ *=* 9.23 (d, *J* = 2.3 Hz, 1H), 8.64 (d, *J* = 2.9 Hz, 1H), 8.47 (s, 1H), 8.17 (d, *J* = 8.4 Hz, 1H), 7.93–7.79 (m, 2H), 7.65 (t, *J* = 7.6 Hz, 1H), 7.17 (t, *J* = 8.1 Hz, 1H), 6.91–6.81 (m, 2H), 6.78 (m, *J* = 8.5, 2.5 Hz, 1H), 3.33–3.20 (m, 4H), 3.05 (t, *J* = 5.5 Hz, 2H), 2.73–2.57 (m, 4H), 2.42 (t, *J* = 5.6 Hz, 2H), 1.63 (m, 4H).

^13^C NMR (126 MHz, CDCl_3_) δ = 152.14, 149.10, 147.05, 135.99, 135.12, 134.14, 132.23, 130.24, 129.63, 129.08, 128.34, 126.53, 119.77, 116.10, 114.16, 58.08, 52.86, 48.28, 43.15, 28.87, 24.68.

HRMS (ESI): *m*/*z* calcd for C_23_H_27_ClN_4_O_2_S [M + H]^+^: 459.1616, found: 459.1603.

The m.p., ^1^H NMR and ^13^C NMR were consistent with those reported [18].

### 4.25 N‐(4‐(4‐(2,3‐dichlorophenyl)piperazin‐1‐yl)butyl)quinoline‐3‐sulfonamide (26)

Compound **26** was prepared from **24** (0.456 g, 1.508 mmol) and **8c** (0.200 g, 0.754 mmol) using BnMe_3_N^+^Cl^‐^ (0.490 g, 2.638 mmol), TCCA (0.210 g, 0.905 mmol) and 1 M Na_2_CO_3_ solution (1.508 mL, 1.508 mmol).

0.258 gYield (70%); White Solid; m.p. 123.8°C–126.0°C.

^1^H NMR (500 MHz, CDCl_3_) δ *=* 9.26 (s, 1H), 8.92 (s, 1H), 8.68 (s, 1H), 8.18 (d, *J* = 8.5 Hz, 1H), 7.92 (d, *J* = 8.1 Hz, 1H), 7.87 (t, *J* = 7.1 Hz, 1H), 7.67 (t, *J* = 7.6 Hz, 1H), 7.18 (m, *J* = 6.9 Hz, 2H), 7.04 (m, *J* = 7.3, 2.4 Hz, 1H), 3.22 (m, *J* = 5.3 Hz, 4H), 3.04 (s, 2H), 2.76 (m, 4H), 2.51 (m, 2H), 1.66 (m, 4H).

^13^C NMR (126 MHz, CDCl_3_) δ = 151.02, 149.09, 147.14, 136.04, 134.19, 134.10, 132.20, 129.67, 129.06, 128.32, 127.77, 127.68, 126.55, 125.05, 119.14, 58.29, 53.27, 50.70, 43.14, 28.97, 24.81.

HRMS (ESI): *m*/*z* calcd for C_23_H_26_Cl_2_N_4_O_2_S [M+H]^+^: 493.1226, found: 493.1227.

The m.p., ^1^H NMR and ^13^C NMR were consistent with those reported [19].

## Supporting Information

Additional supporting information can be found online in the Supporting Information section.

## Funding

This research was funded by Special Funds of National Natural Science Foundation of China (grant number 82441046), Zhongshan Municipal Natural Science Foundation (grant number 2023B2019), and Creative Research Group of Zhongshan City (Lingnan Pharmaceutical Research and Innovation team) (grant number CXTD2022011).

## Conflicts of Interest

The authors declare no conflicts of interest.

## Supporting information

Supplementary Material

## Data Availability

The data that support the findings of this study are available in the Supporting Information of this article.
