# Supplementary material for: A Convenient One‐Pot CuI‐Catalyzed Procedure for the Synthesis of S‐(N‐Heteroaryl) Thiocarboxylates From N‐Heteroaryl Bromides and Thiobenzoic Acid
Source: ChemistryOpen. 2026 Mar 29;15(4):e70186. doi: 10.1002/open.70186 (PMC13140705; doi:10.1002/open.70186)
Supplement: Supplementary file 1 — Supplementary Material [file OPEN-15-e70186-s001.pdf]

A Convenient One-Pot CuI-Catalyzed Procedure for the Synthesis of *S*-(*N*-Heteroaryl) Thiocarboxylates from *N*-Heteroaryl Bromides and Thiobenzoic Acid

Jiamin Pan[1,2], Suting Xie[1,2], Yong Li[1,2], Jiang Wu[4], Guilong Zhao[1,2,3,*]

[1]School of Pharmaceutical Sciences, Southern Medical University, Guangzhou 510515, China.

[2]Zhongshan Institute for Drug Discovery, Shanghai Institute of Materia Medica, Chinese Academy of Sciences, Zhongshan 528400, China.

[3]Shanghai Institute of Materia Medica, Chinese Academy of Sciences, Shanghai 201203, China.

[4]College of Pharmacy, Shenzhen Technology University, Shenzhen 518118, China.

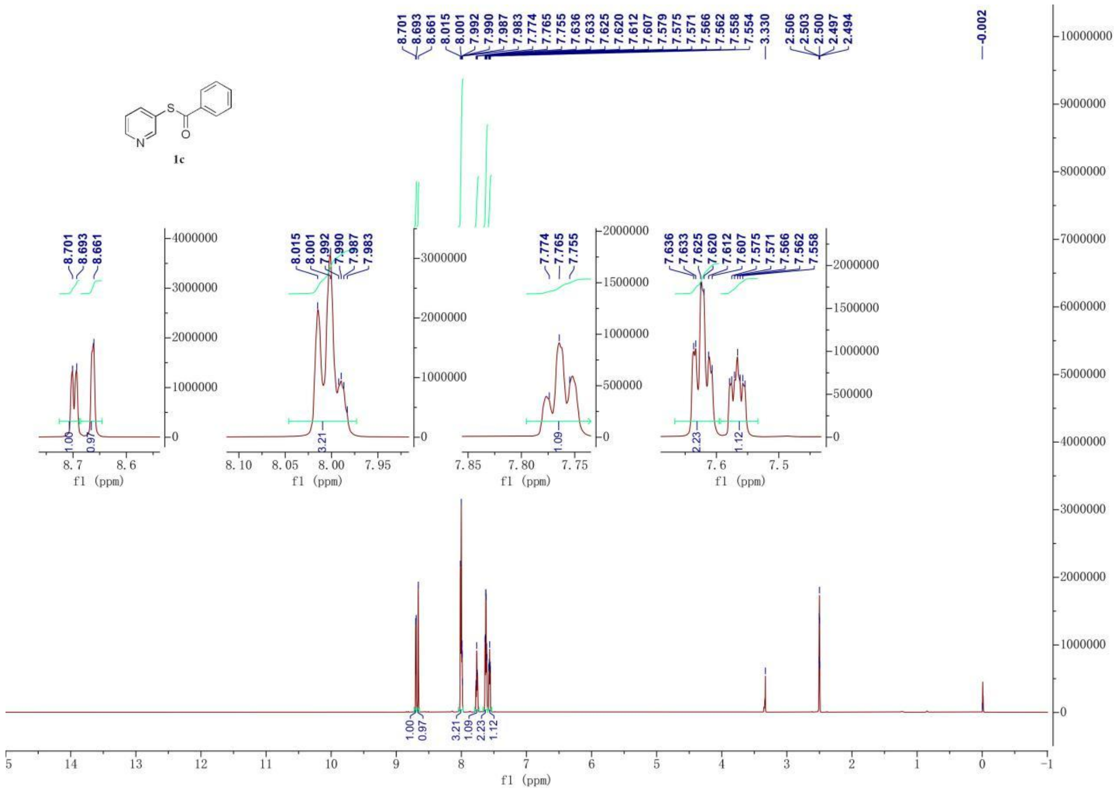

^{1}H NMR (600 MHz, DMSO-d_6) spectrum of **1c**

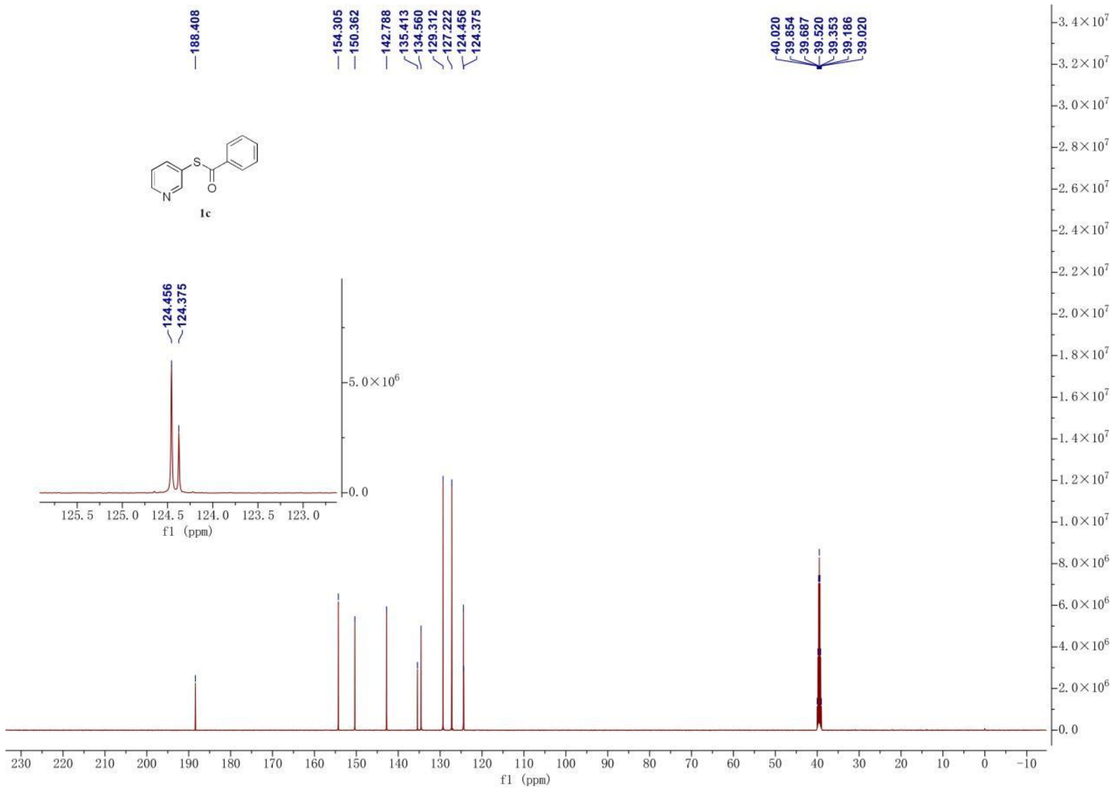

^{13}C NMR (126 MHz, DMSO-d_6) spectrum of **1c**

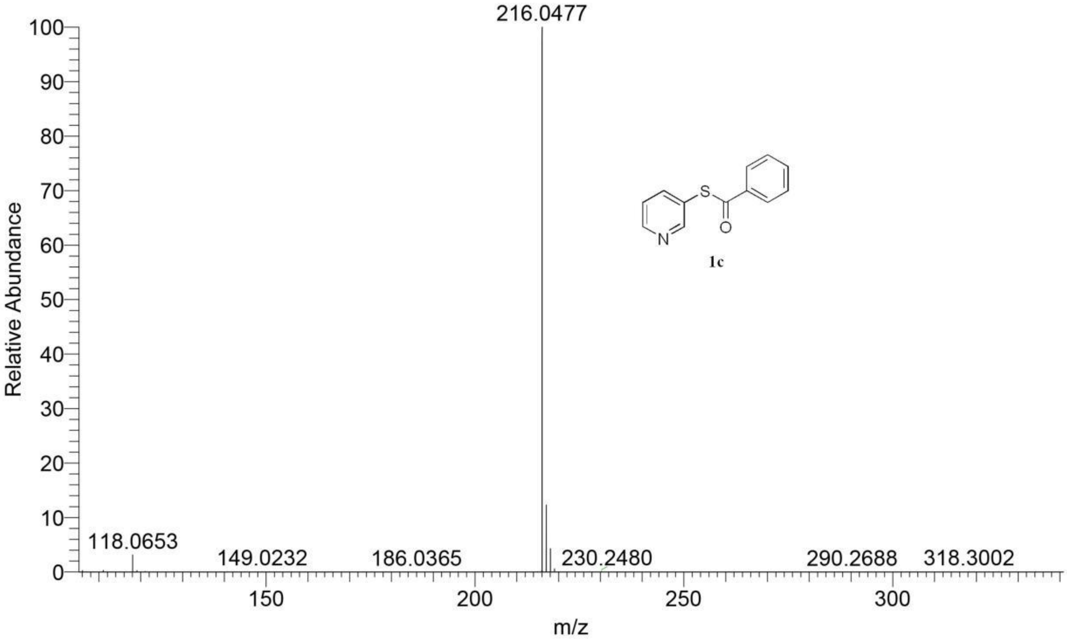

HRMS spectrum of **1c**

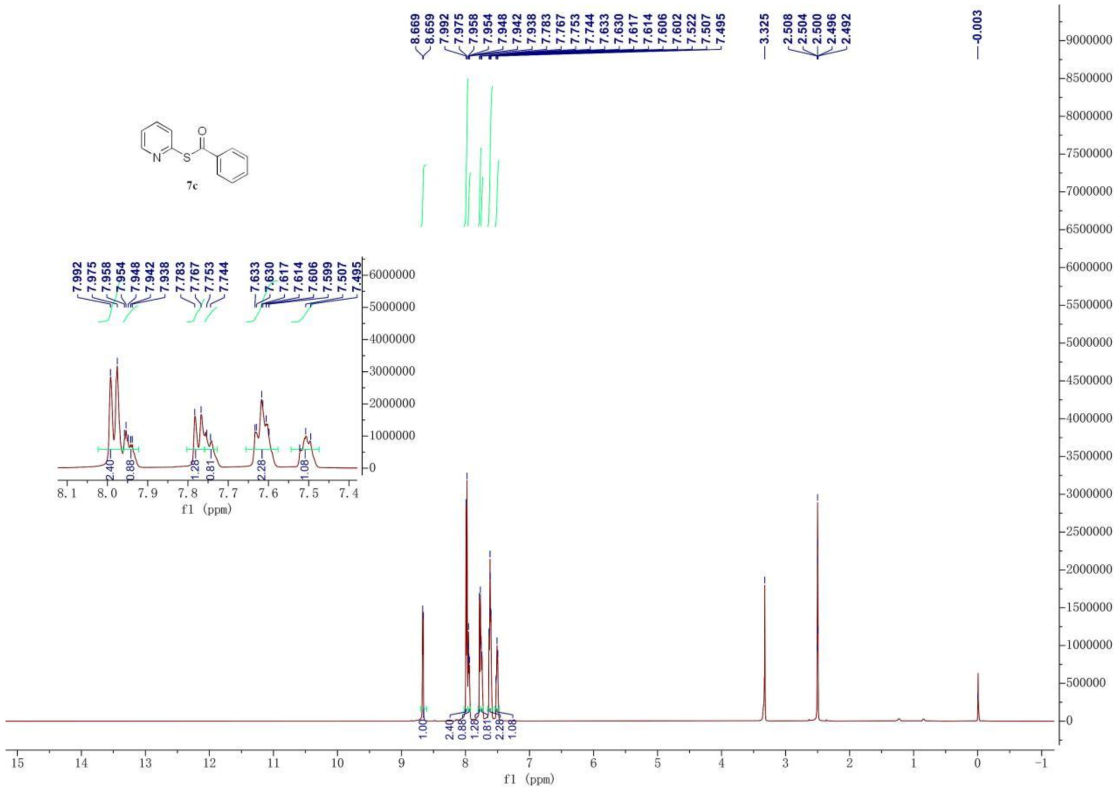

^{1}H NMR (500 MHz, DMSO-d_6) spectrum of **7c**

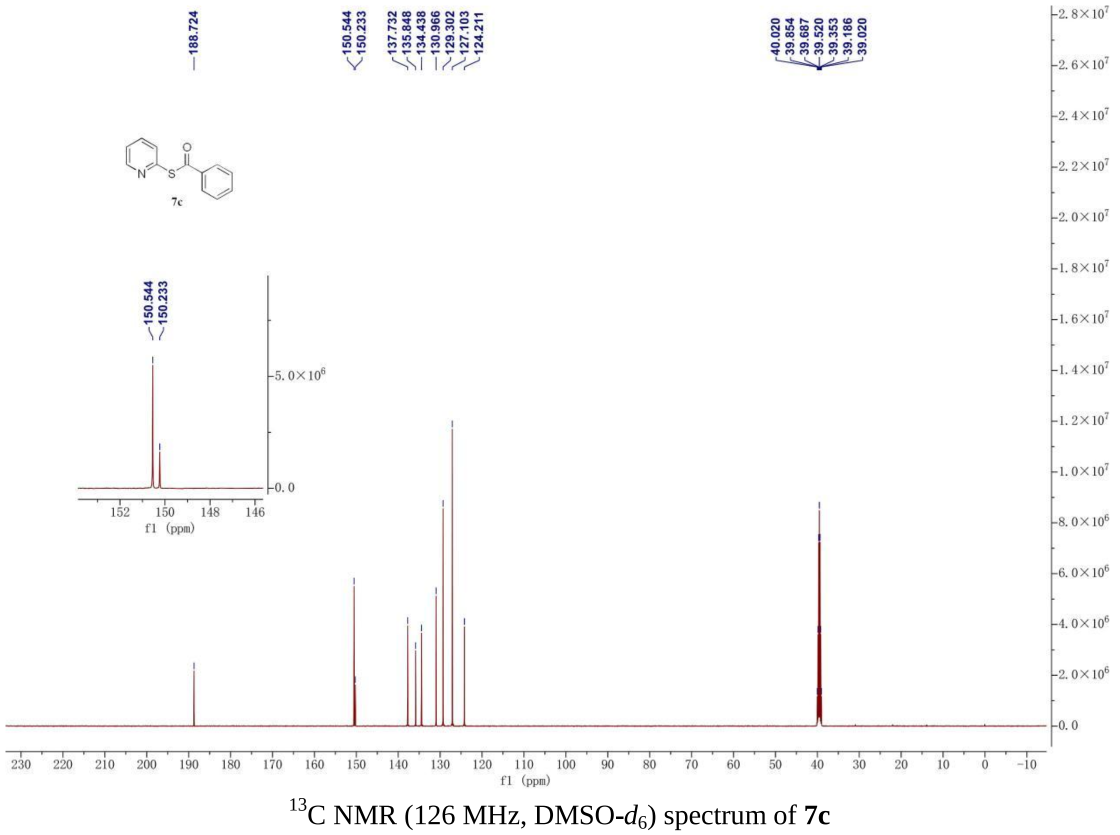

^{13}C NMR (126 MHz, DMSO-d_6) spectrum of **7c**

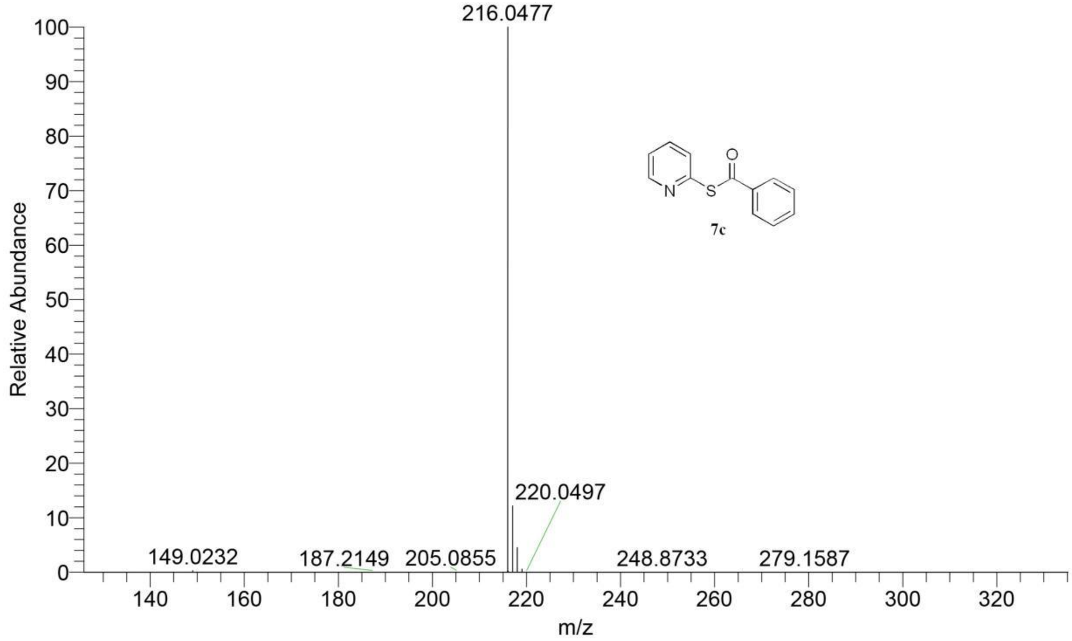

HRMS spectrum of **7c**

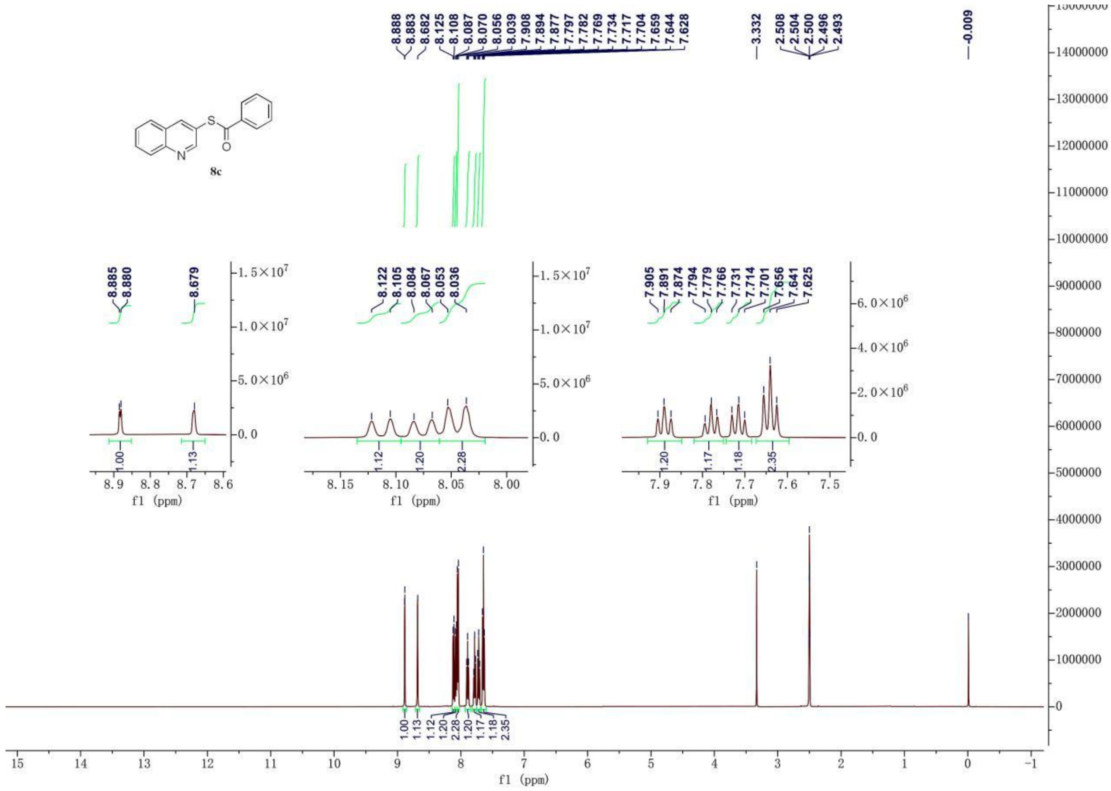

^{1}H NMR (500 MHz, DMSO-d_6) spectrum of **8c**

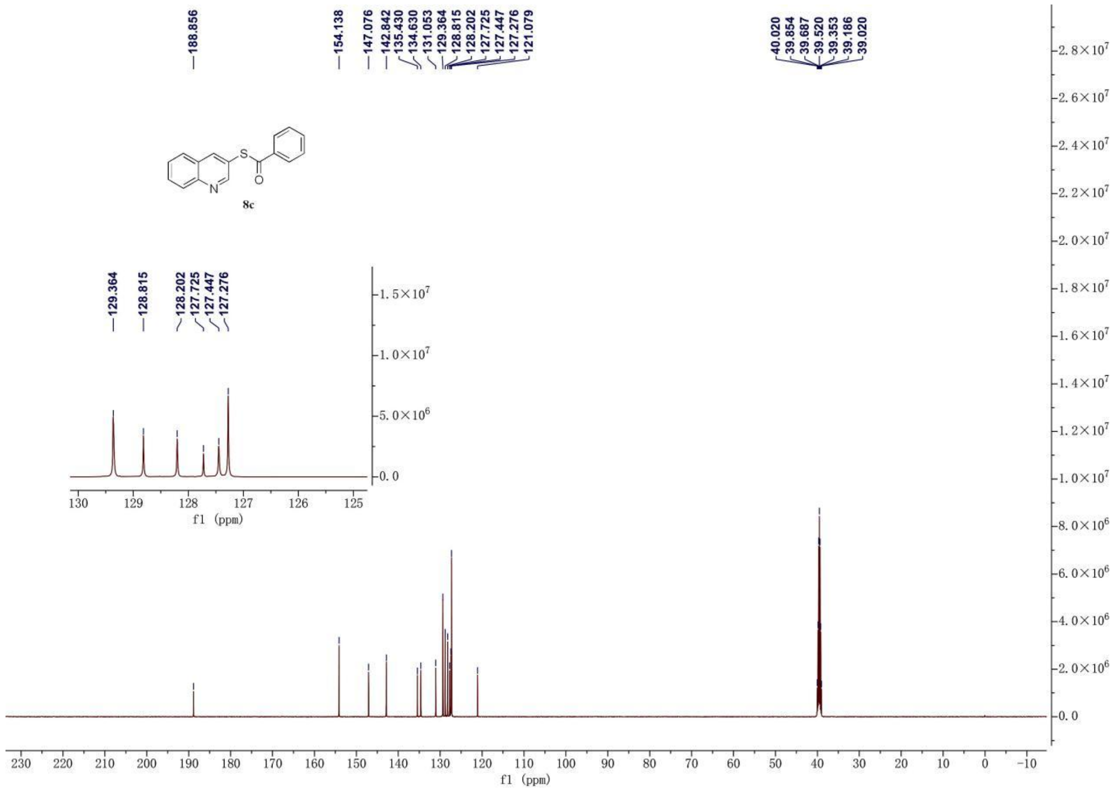

^{13}C NMR (126 MHz, DMSO-d_6) spectrum of **8c**

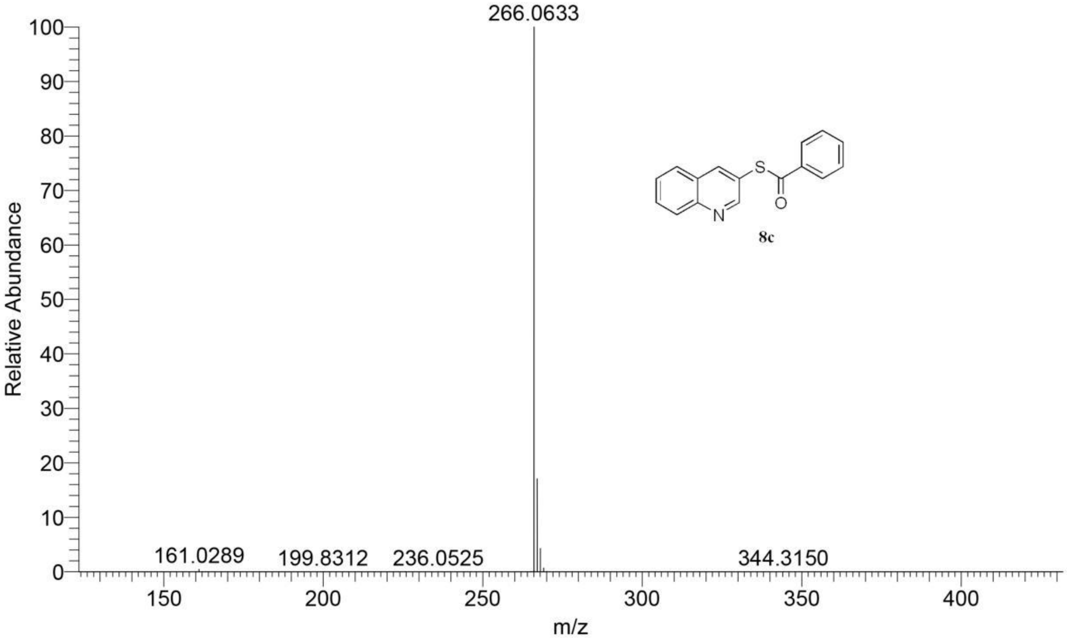

HRMS spectrum of **8c**

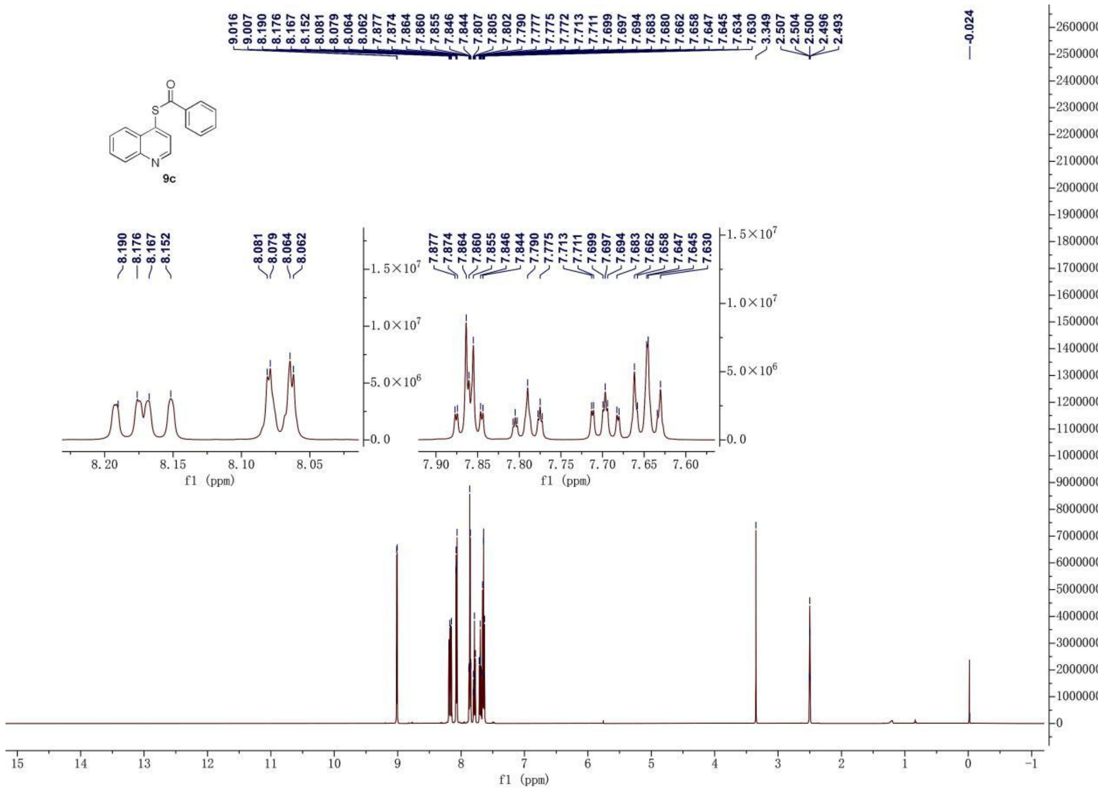

^{1}H NMR (500 MHz, DMSO-d_6) spectrum of **9c**

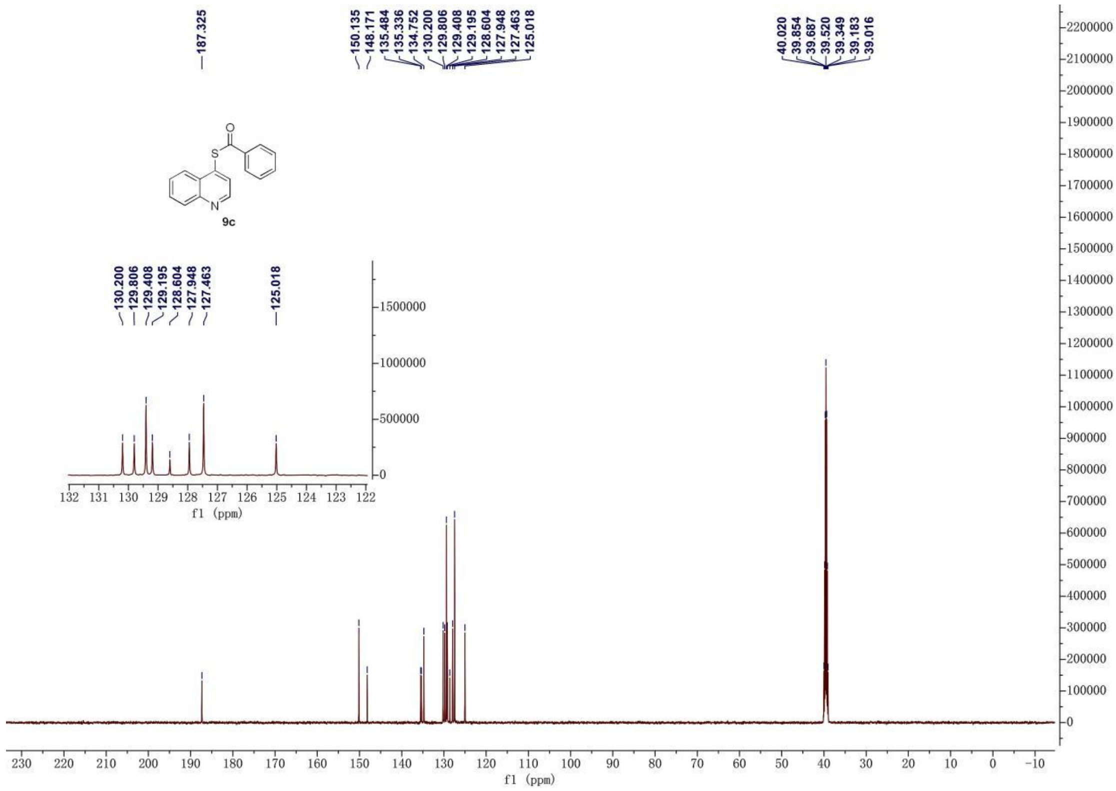

^{13}C NMR (126 MHz, DMSO-d_6) spectrum of **9c**

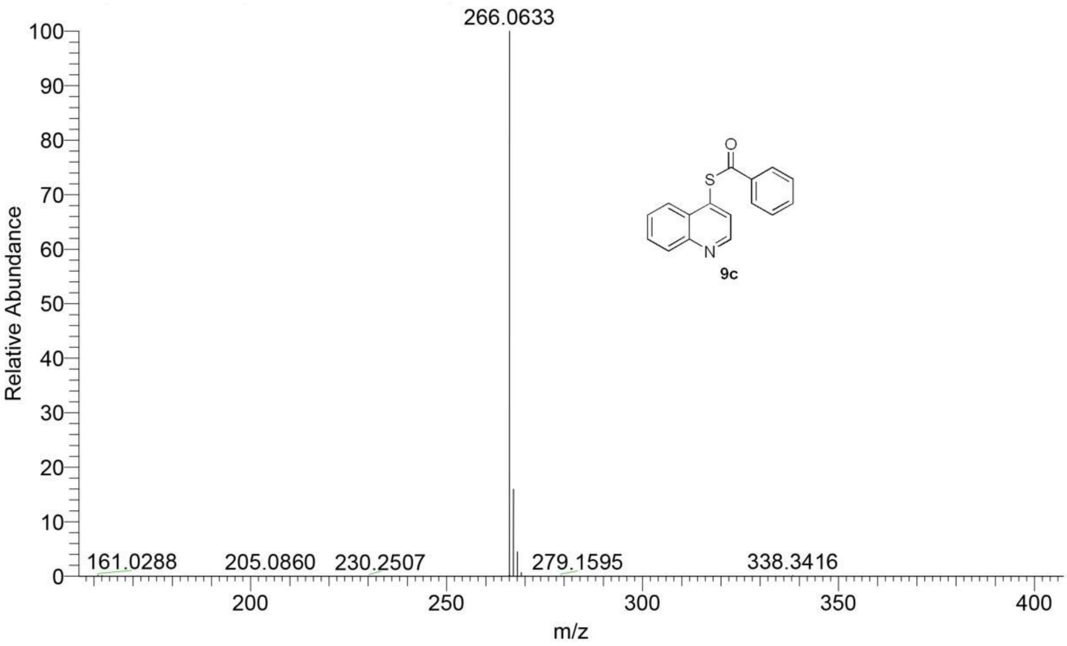

HRMS spectrum of **9c**

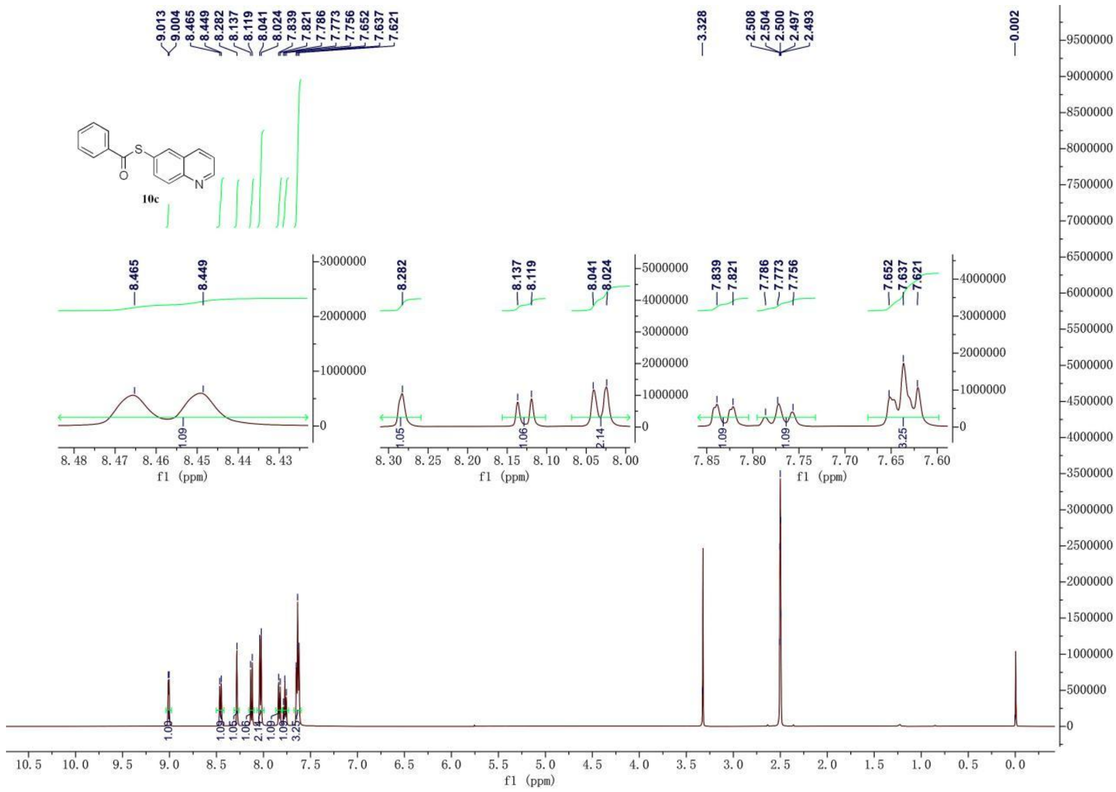

^{1}H NMR (500 MHz, DMSO-d_6) spectrum of **10c**

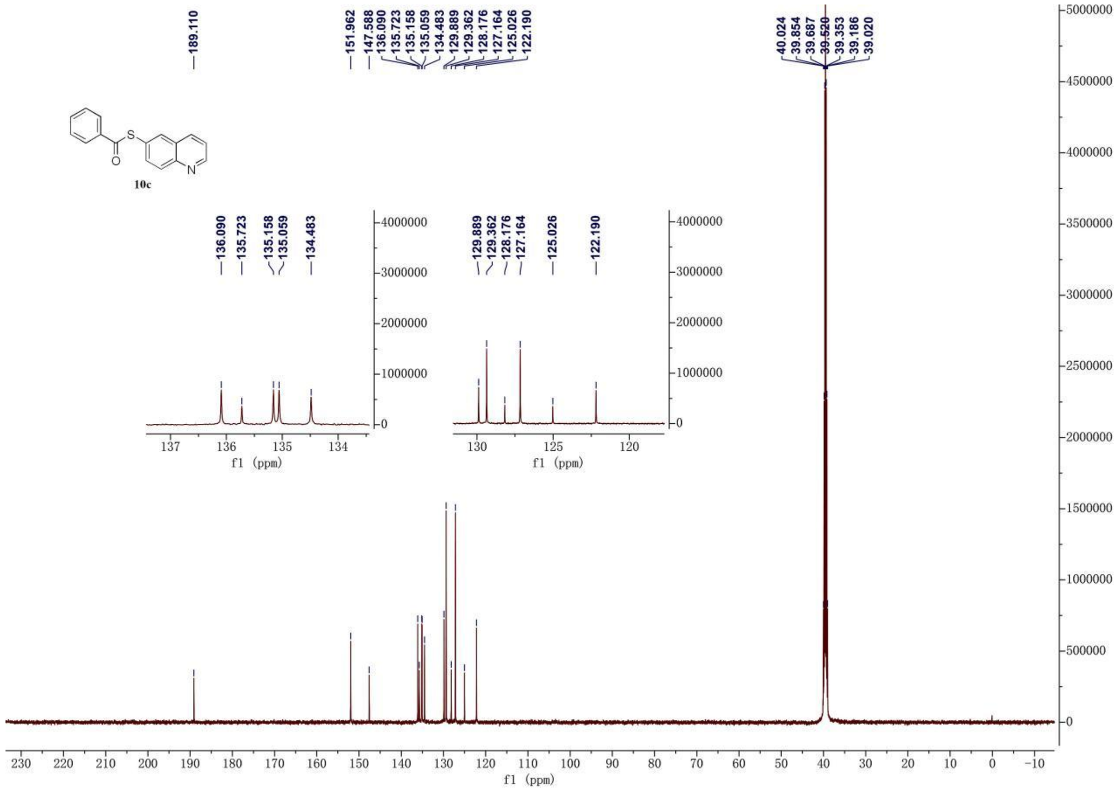

^{13}C NMR (126 MHz, DMSO-d_6) spectrum of **10c**

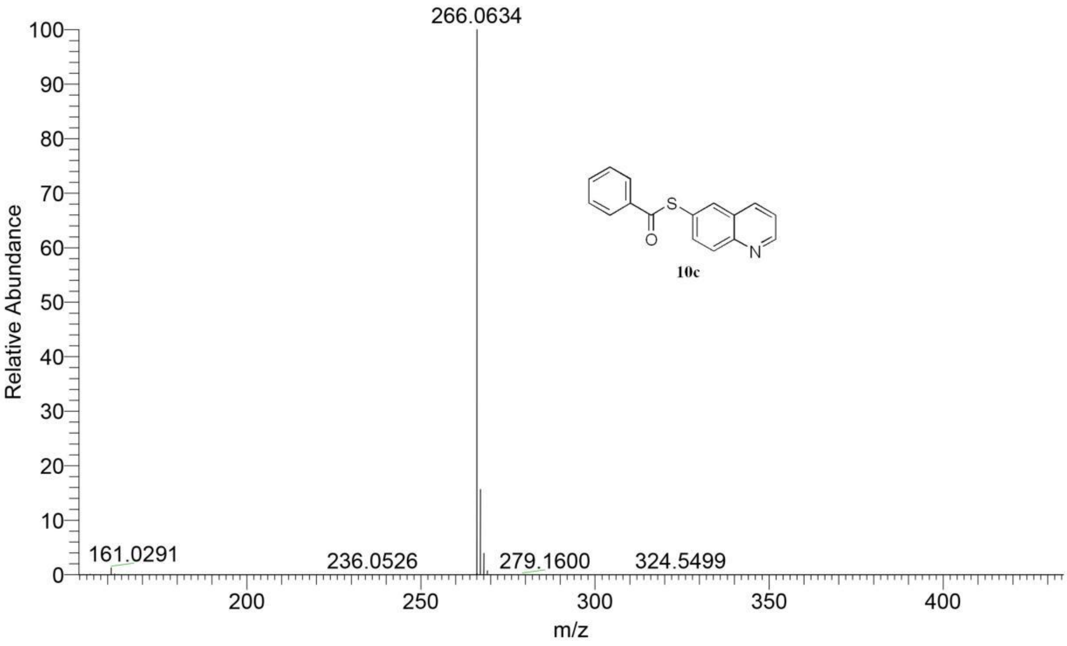

HRMS spectrum of **10c**

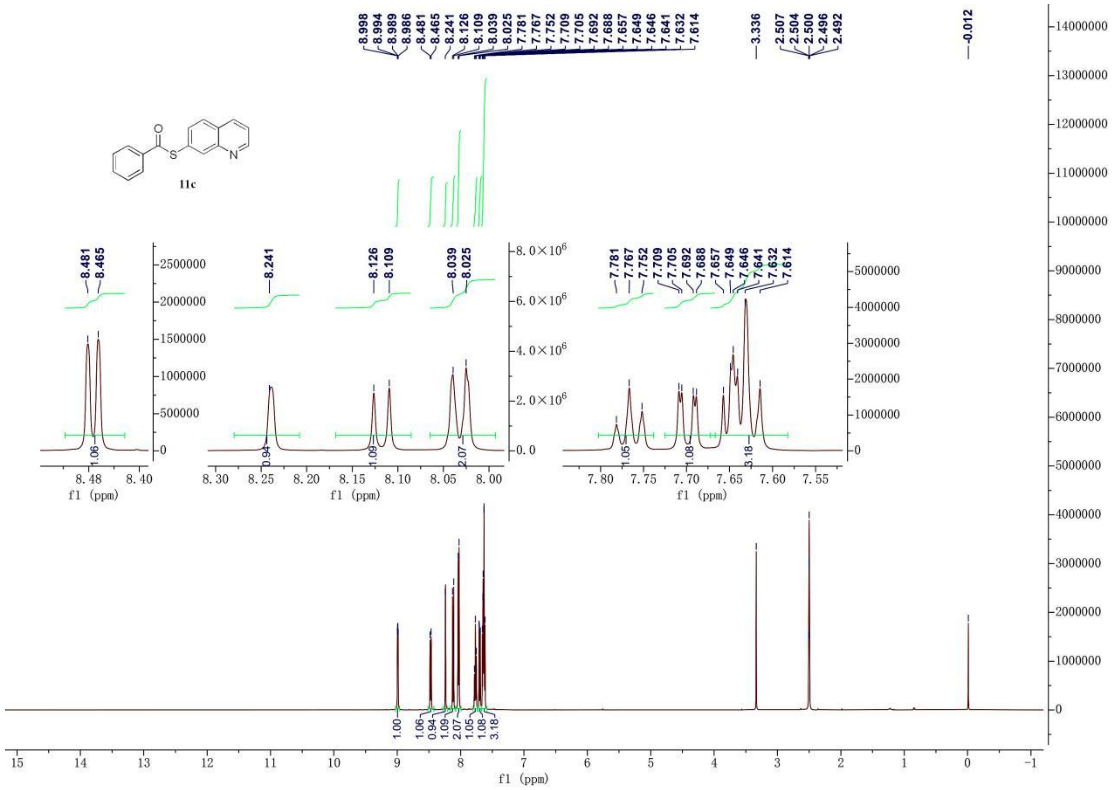

^{1}H NMR (500 MHz, DMSO-d_6) spectrum of **11c**

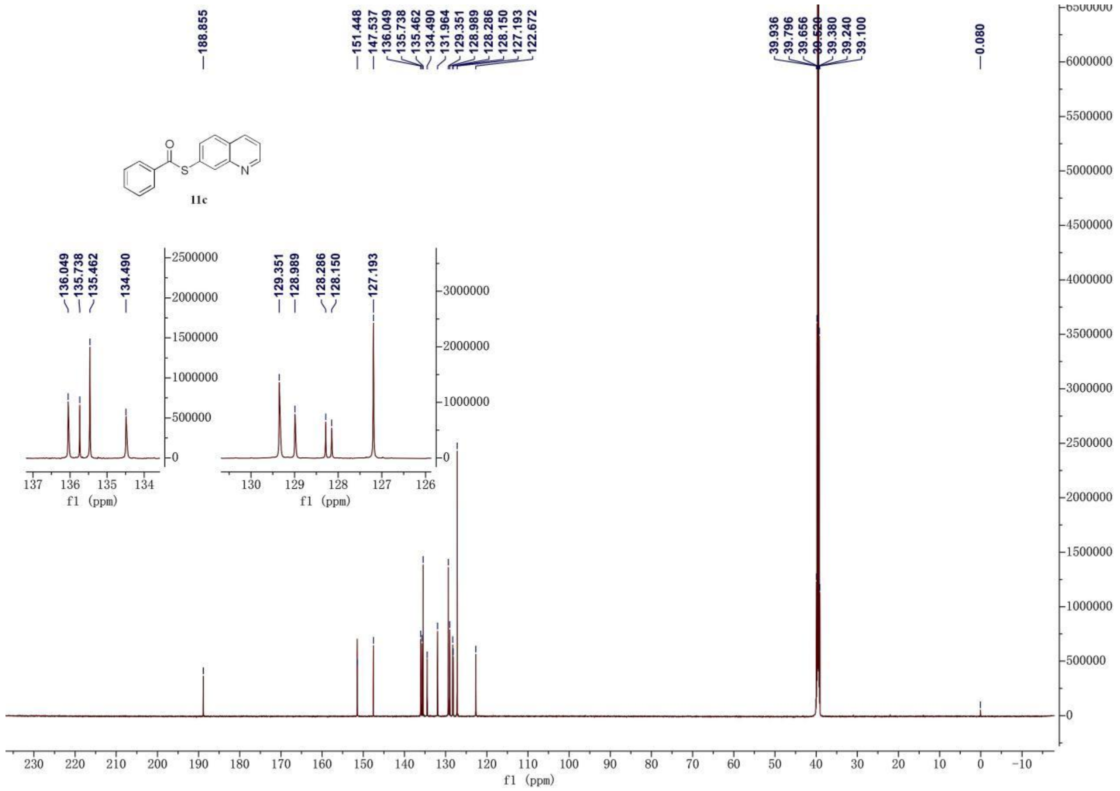

^{13}C NMR (126 MHz, DMSO-d_6) spectrum of **11c**

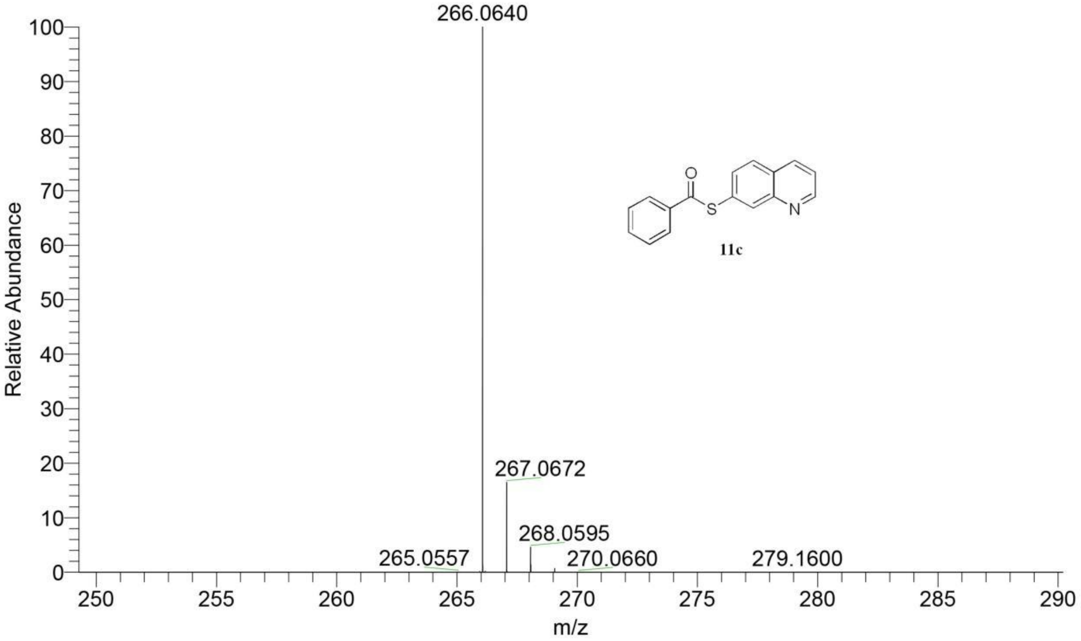

HRMS spectrum of **11c**

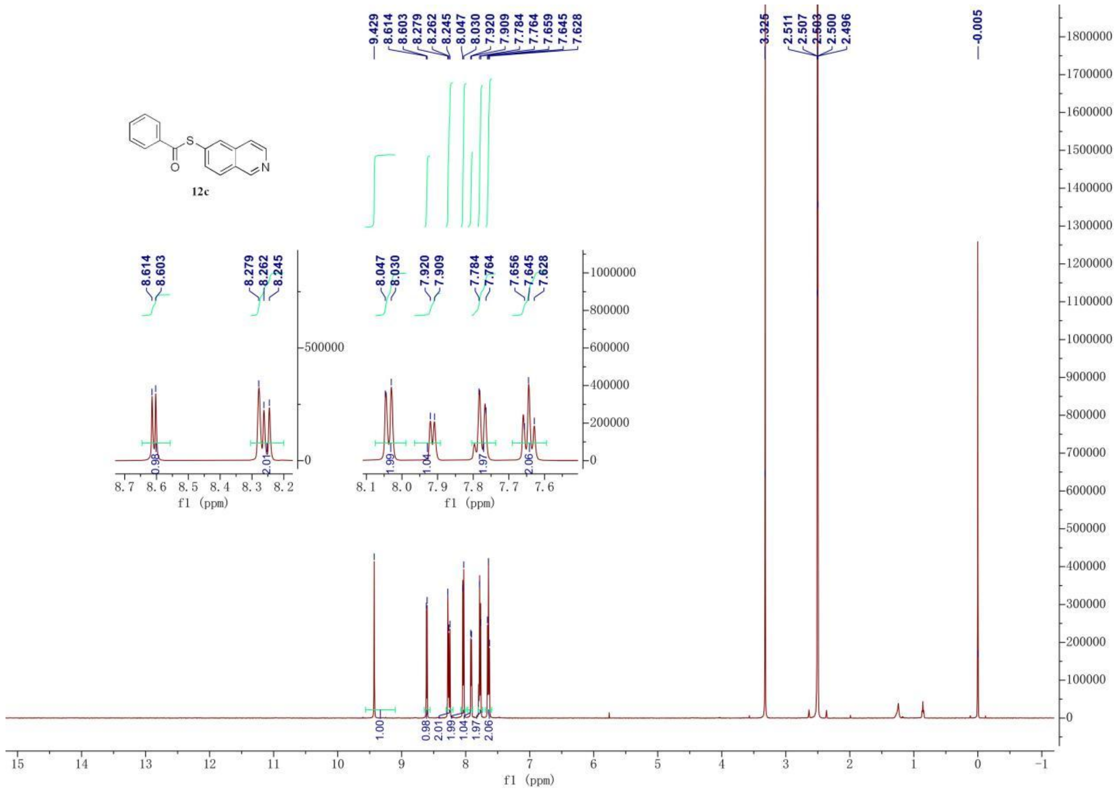

^{1}H NMR (500 MHz, DMSO-d_6) spectrum of **12c**

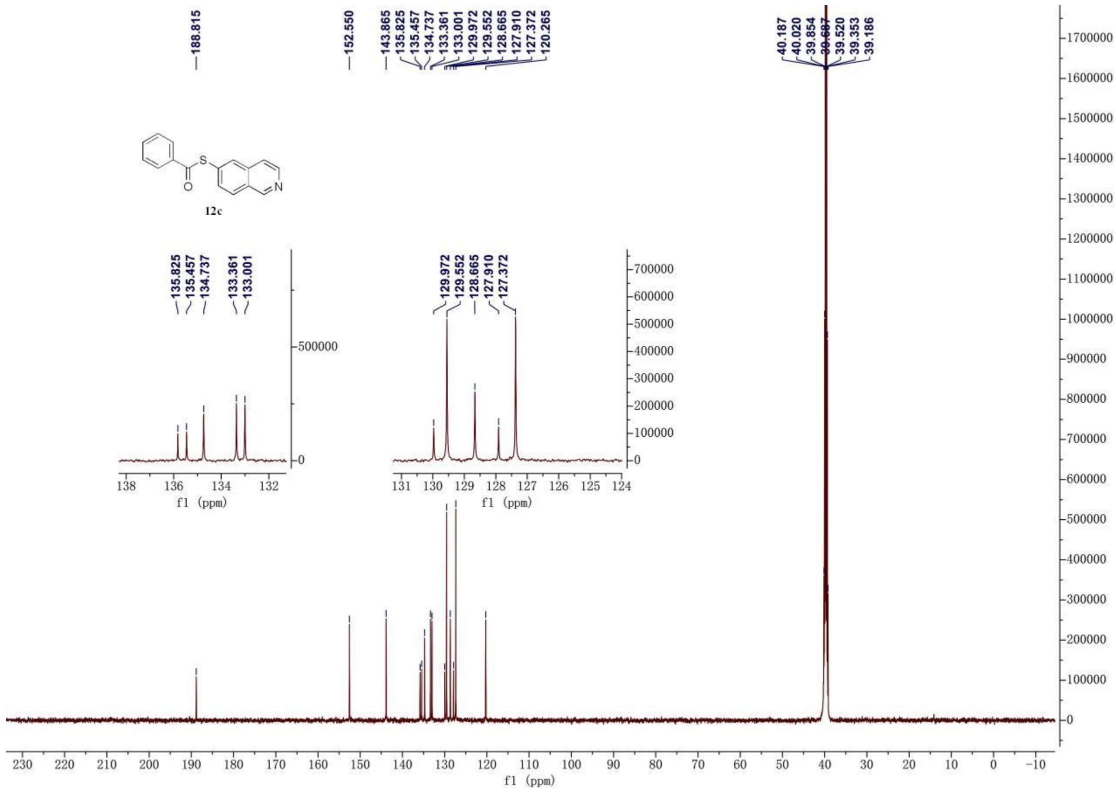

^{13}C NMR (126 MHz, DMSO-d_6) spectrum of **12c**

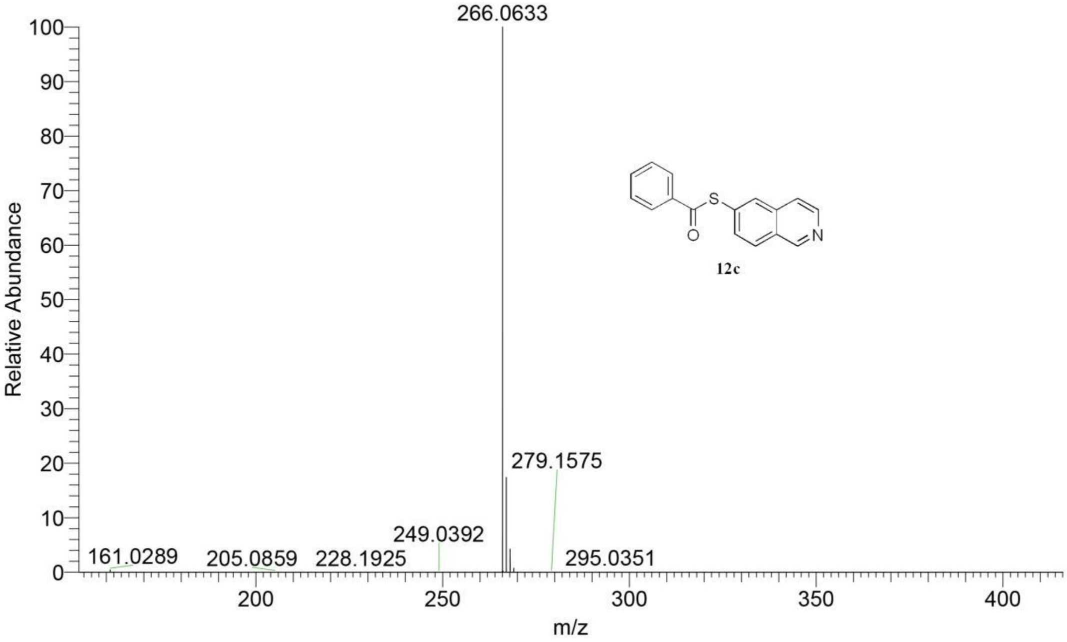

HRMS spectrum of **12c**

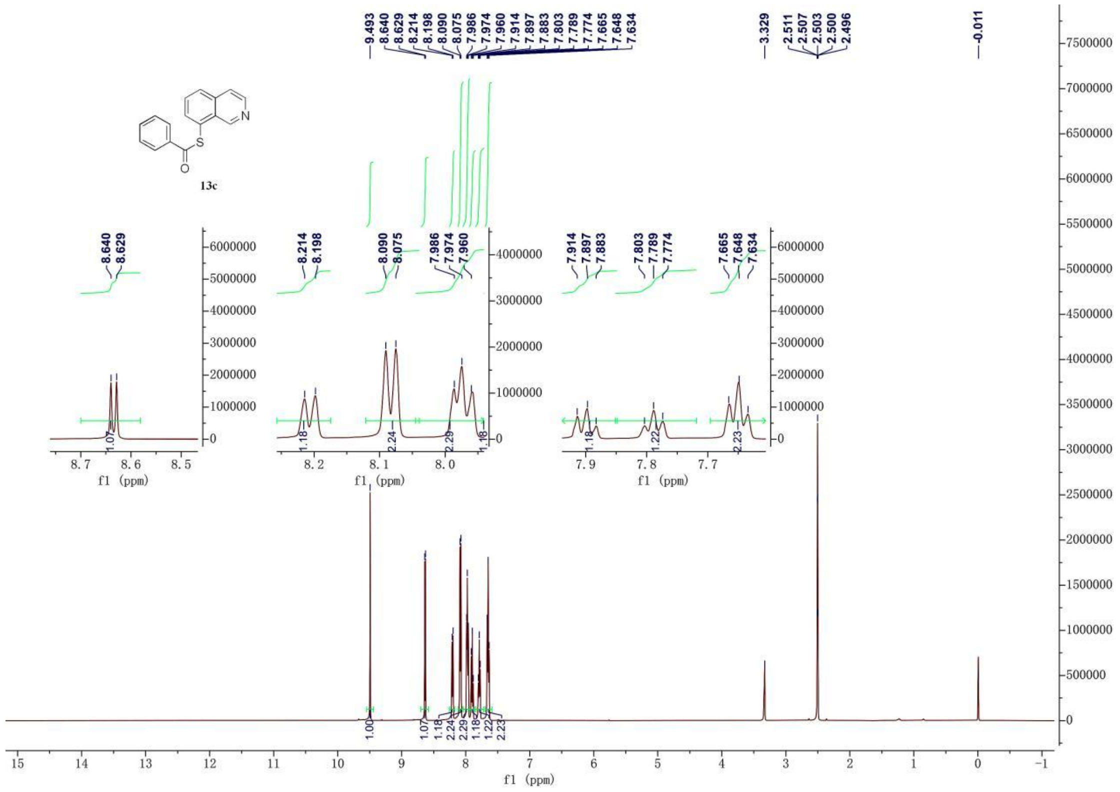

^{1}H NMR (500 MHz, DMSO-d_6) spectrum of **13c**

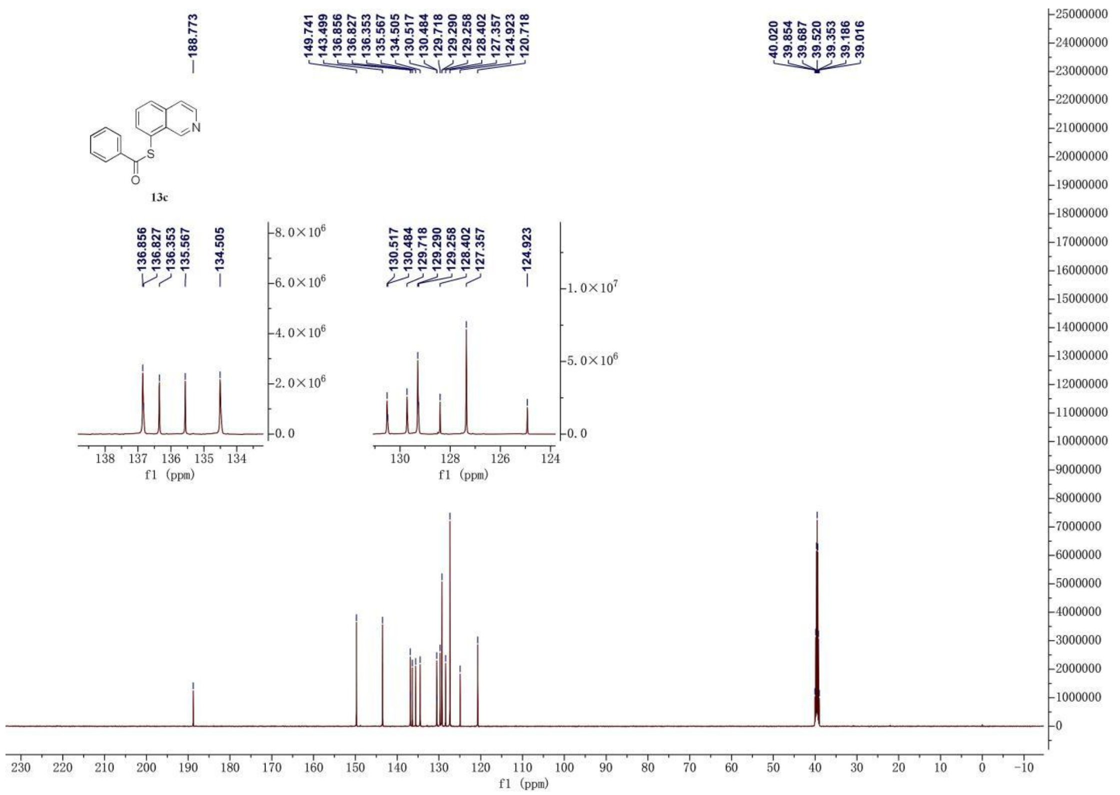

^{13}C NMR (126 MHz, DMSO-d_6) spectrum of **13c**

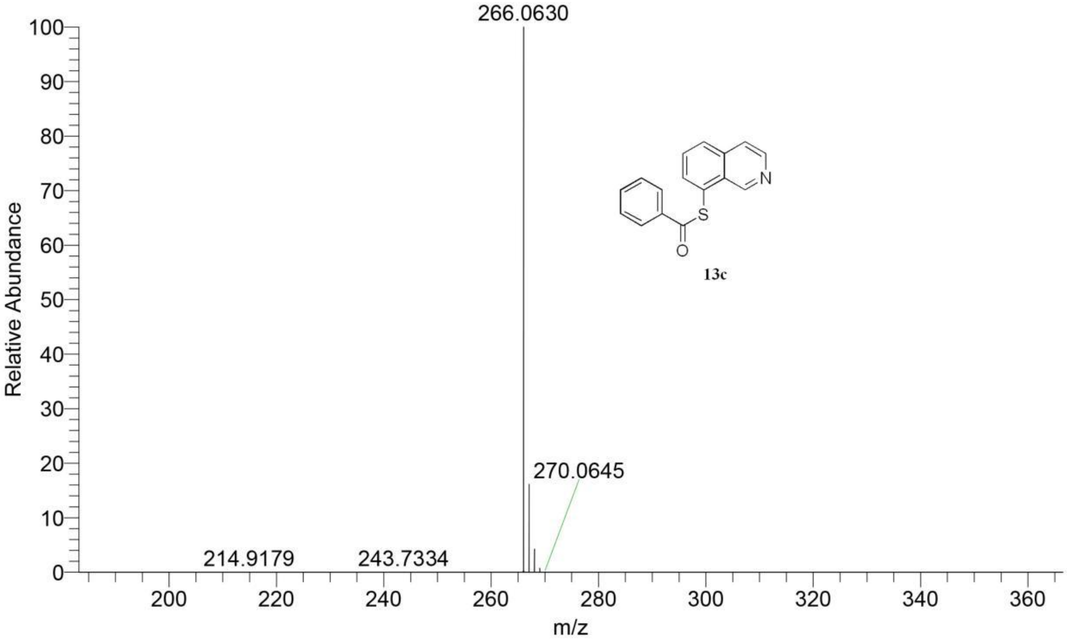

HRMS spectrum of **13c**

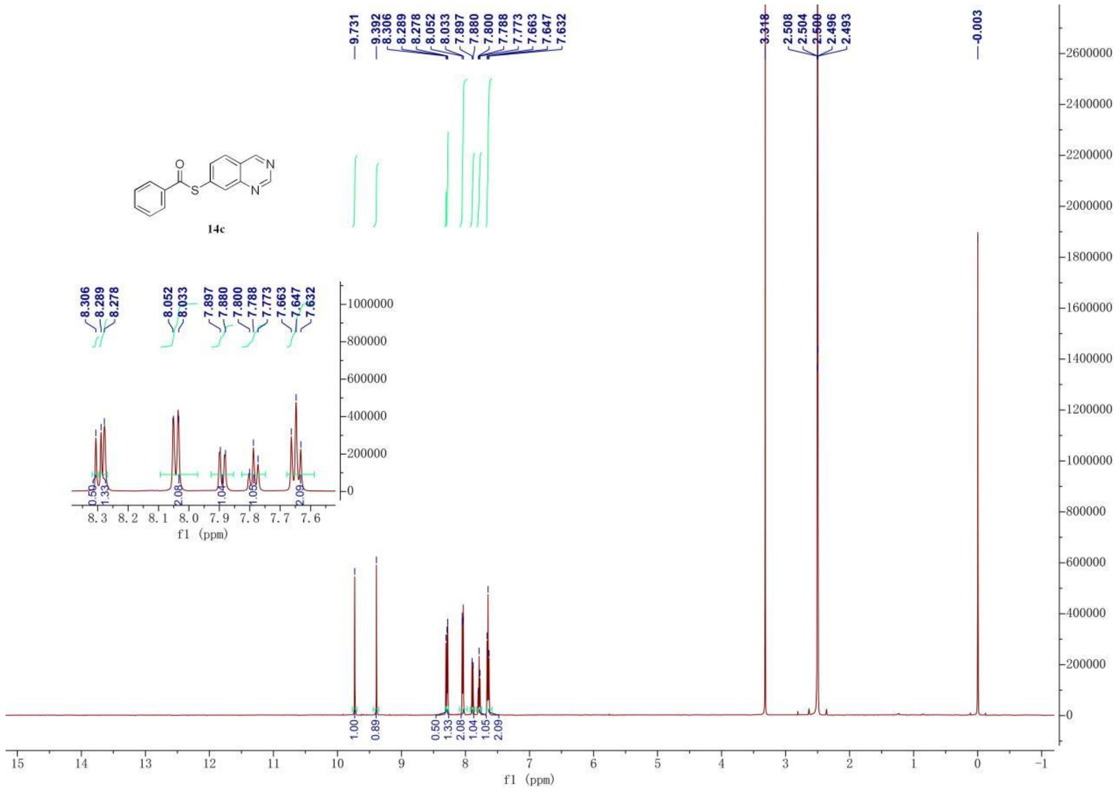

^{1}H NMR (500 MHz, DMSO-d_6) spectrum of **14c**

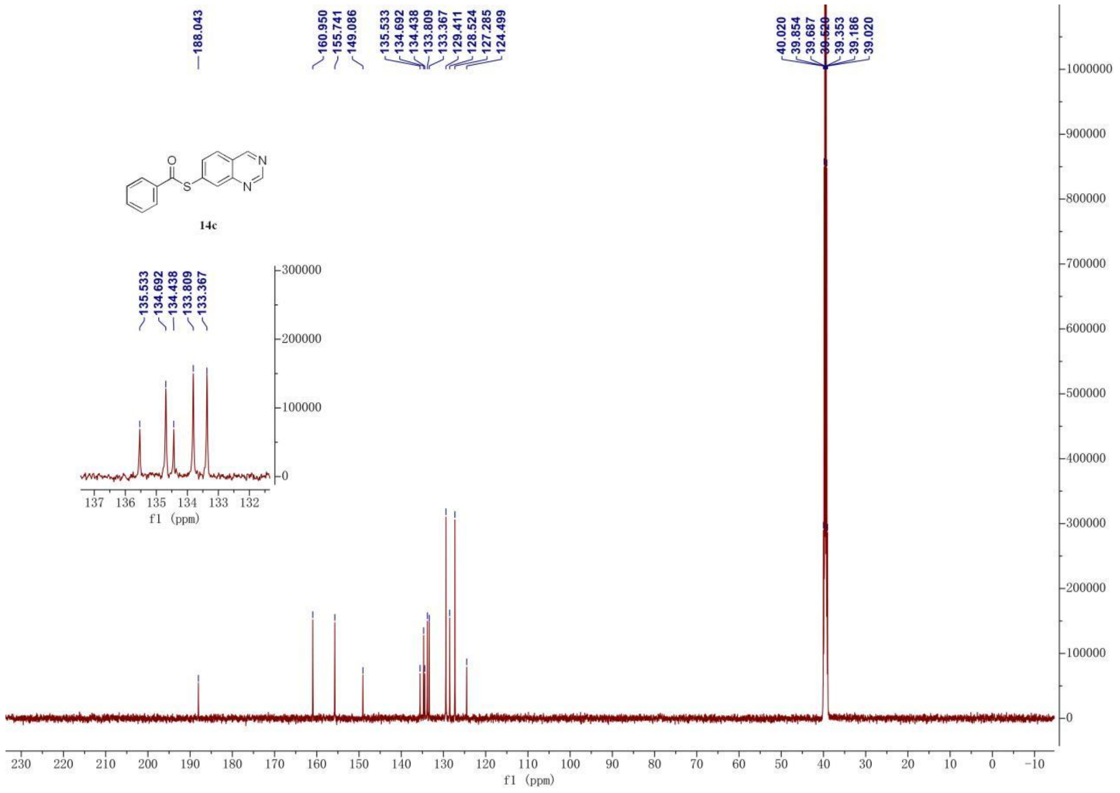

^{13}C NMR (126 MHz, DMSO-d_6) spectrum of **14c**

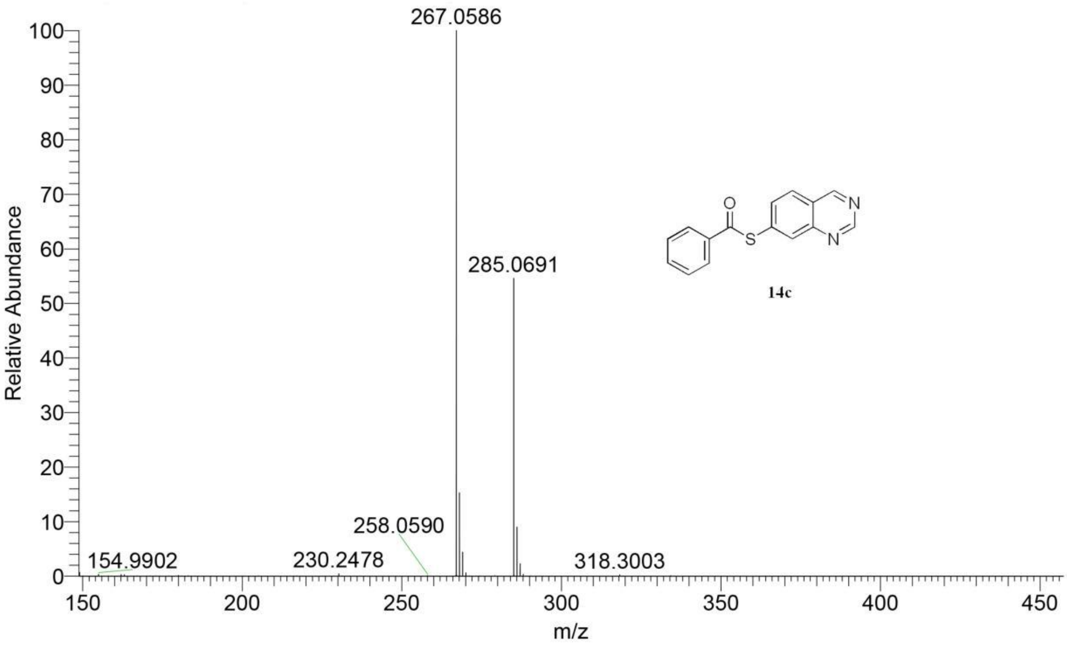

HRMS spectrum of **14c**

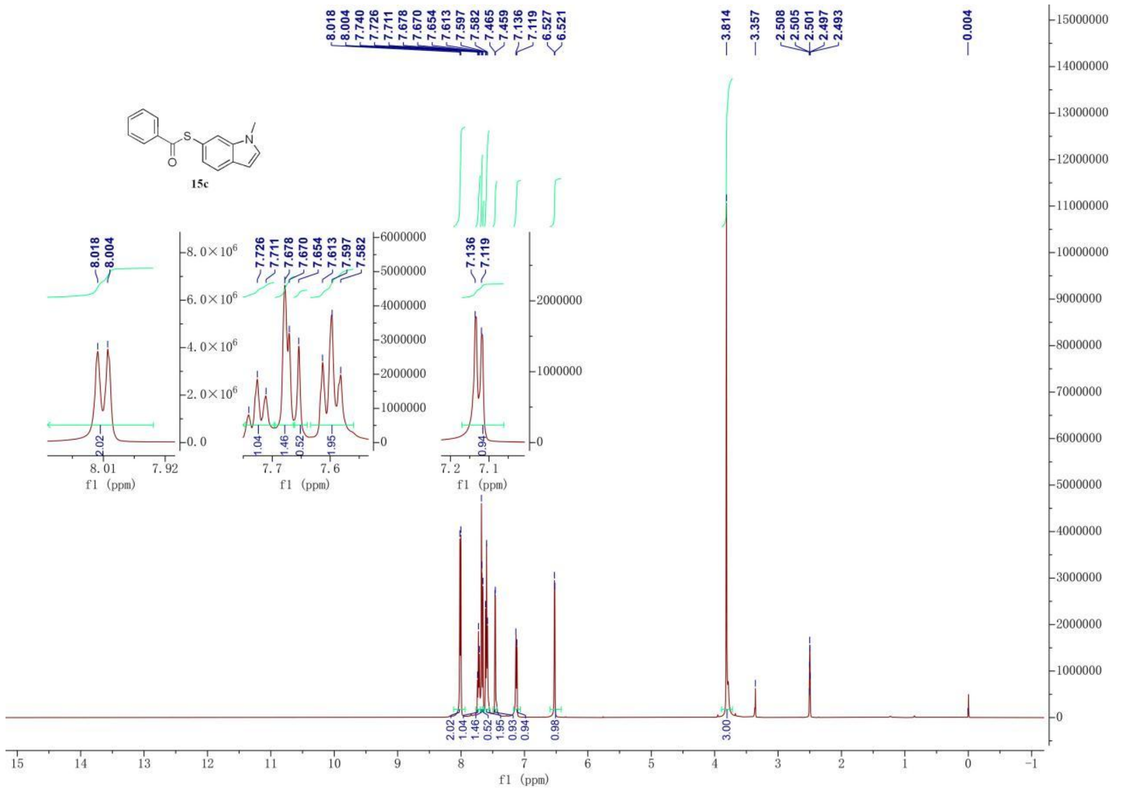

^{1}H NMR (500 MHz, DMSO-d_6) spectrum of **15c**

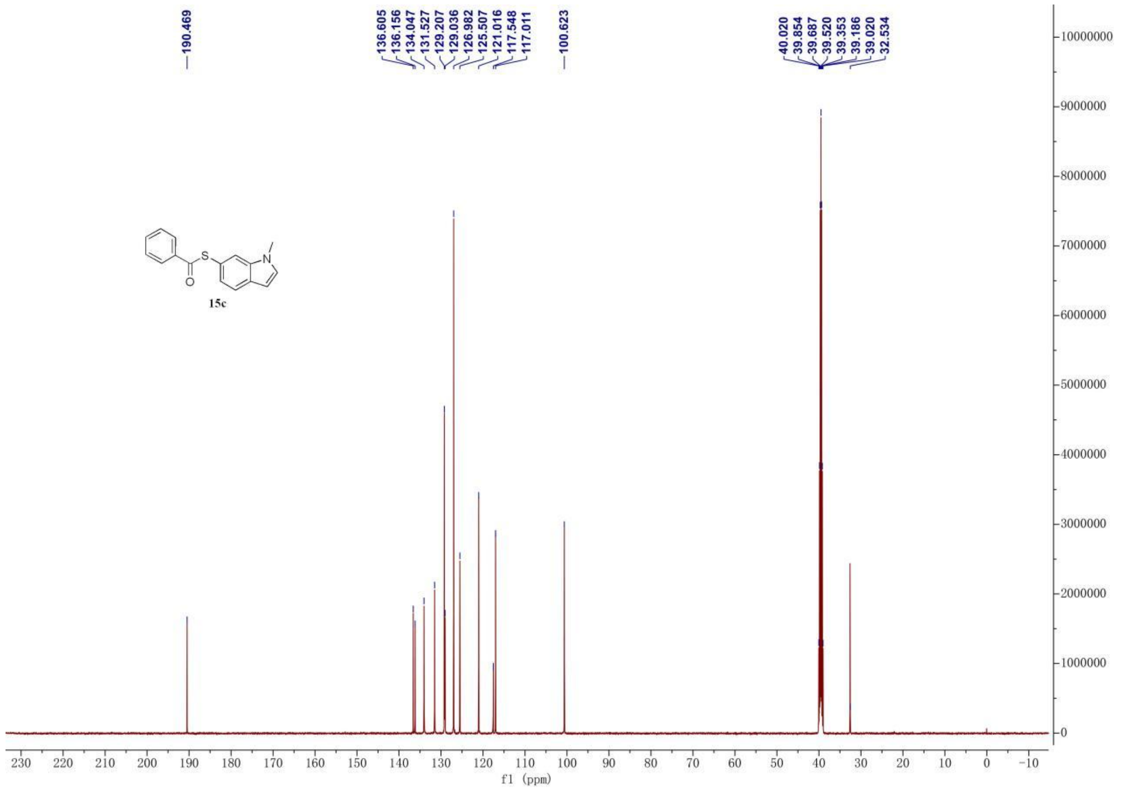

^{13}C NMR (126 MHz, DMSO-d_6) spectrum of **15c**

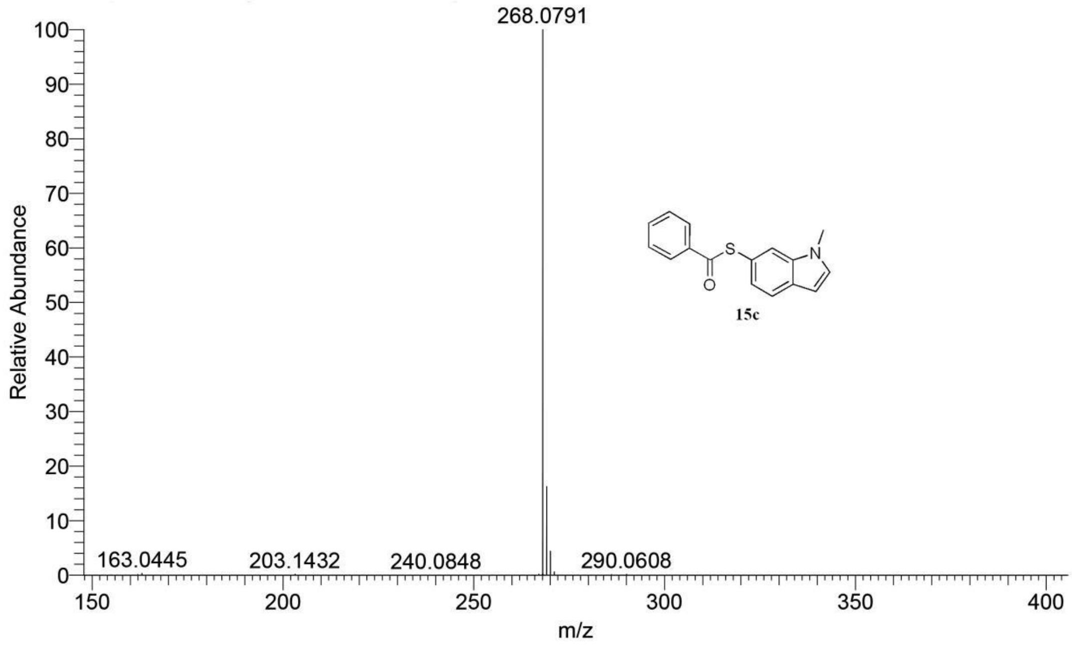

HRMS spectrum of **15c**

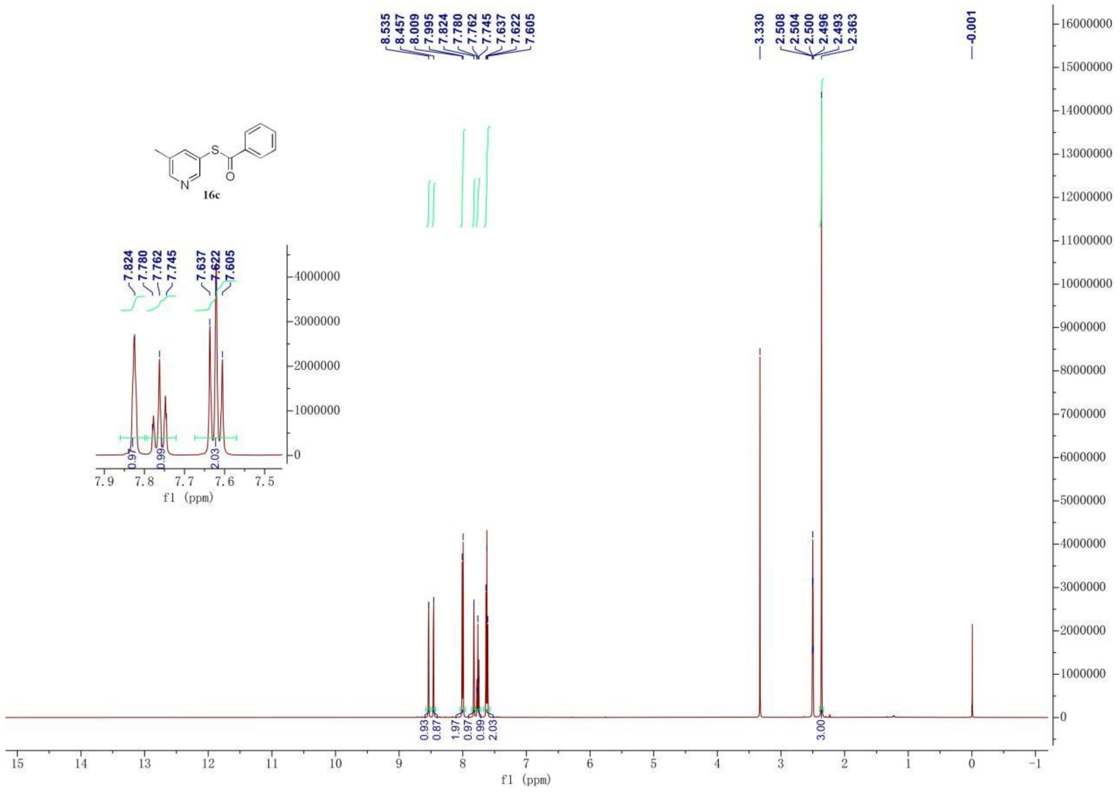

^{1}H NMR (500 MHz, DMSO-d_6) spectrum of **16c**

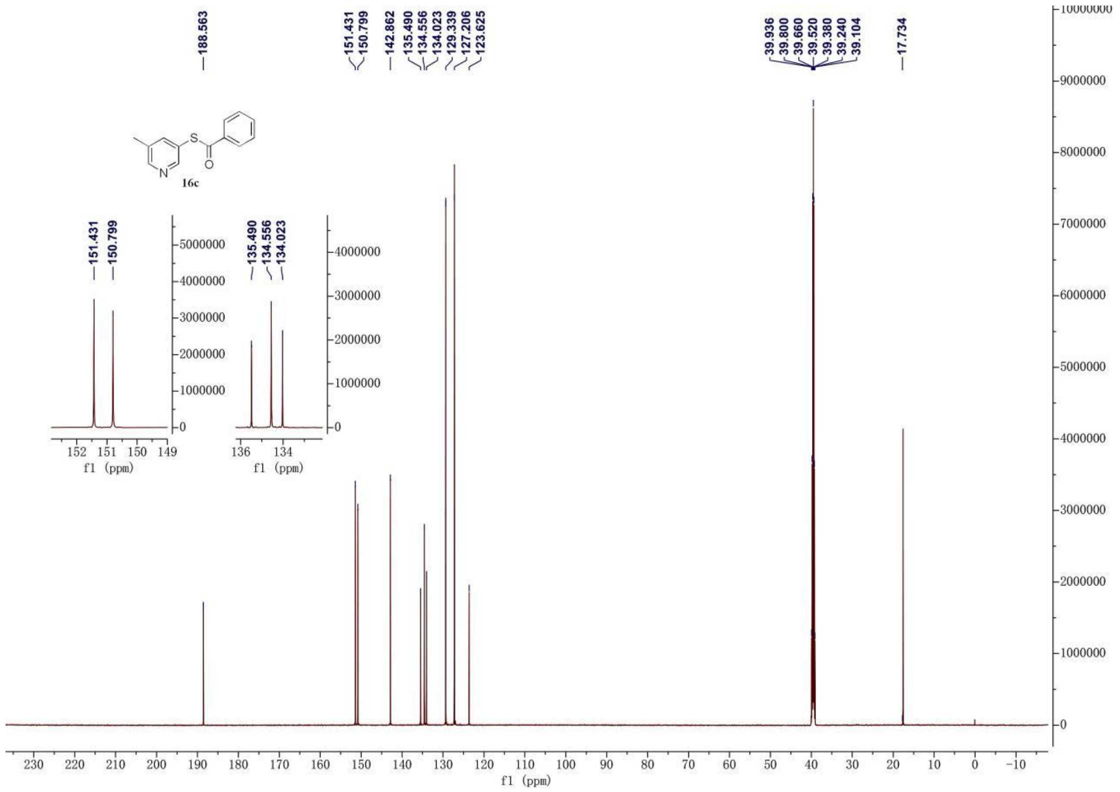

^{13}C NMR (151 MHz, DMSO-d_6) spectrum of **16c**

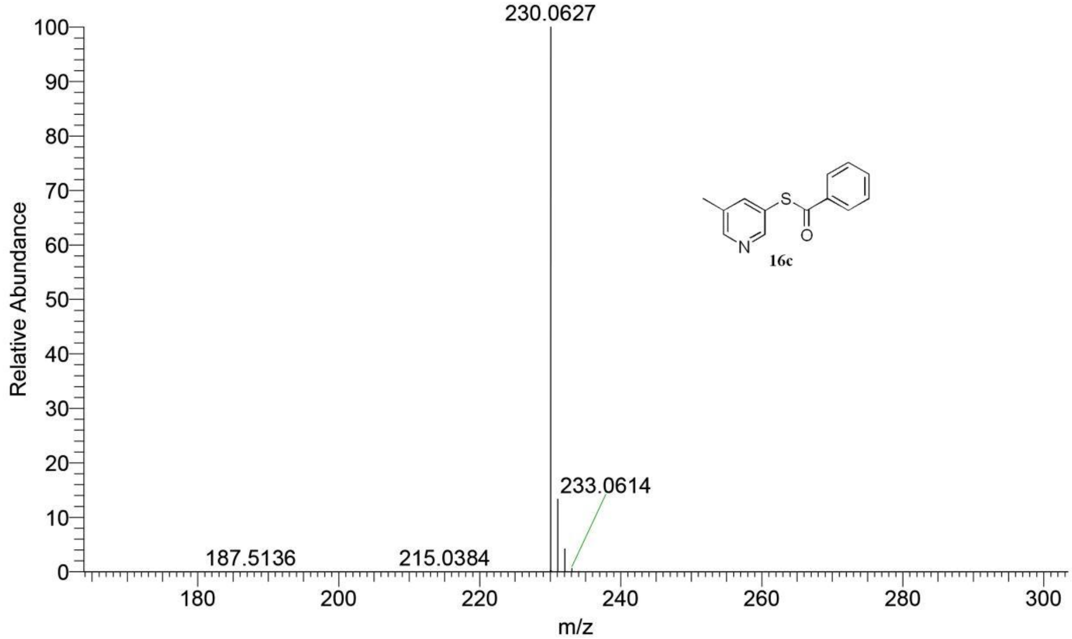

HRMS spectrum of **16c**

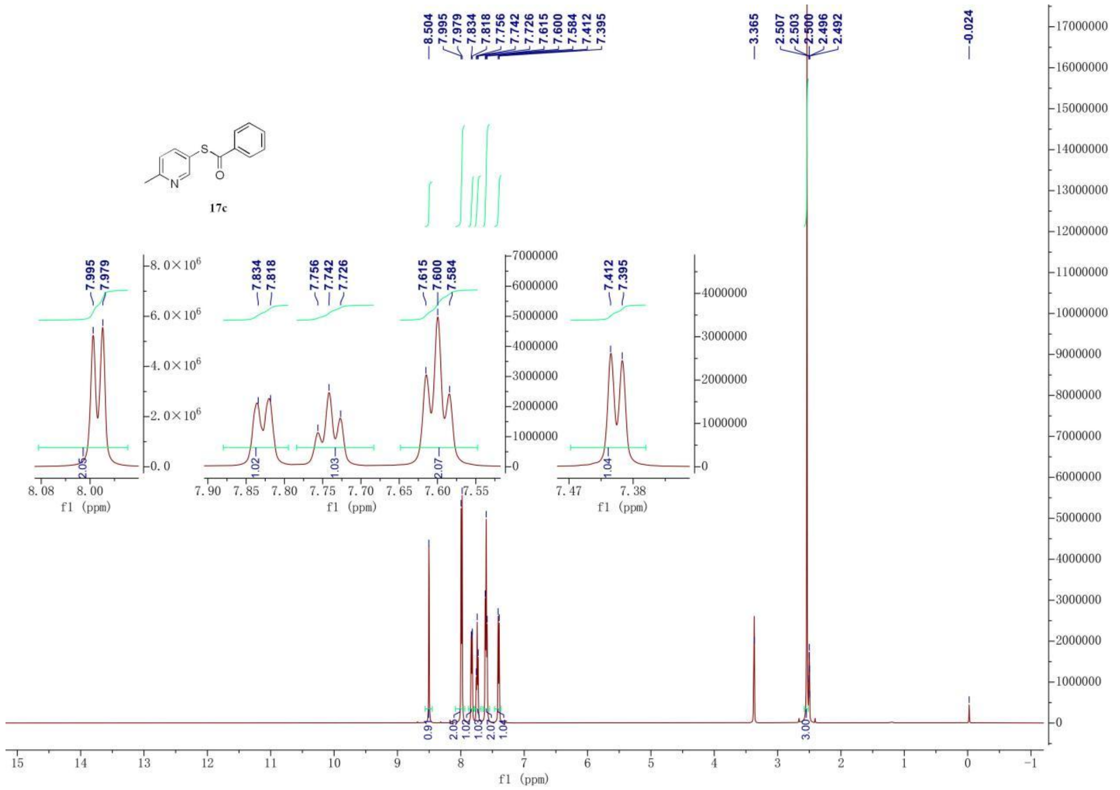

^{1}H NMR (500 MHz, DMSO-d_6) spectrum of **17c**

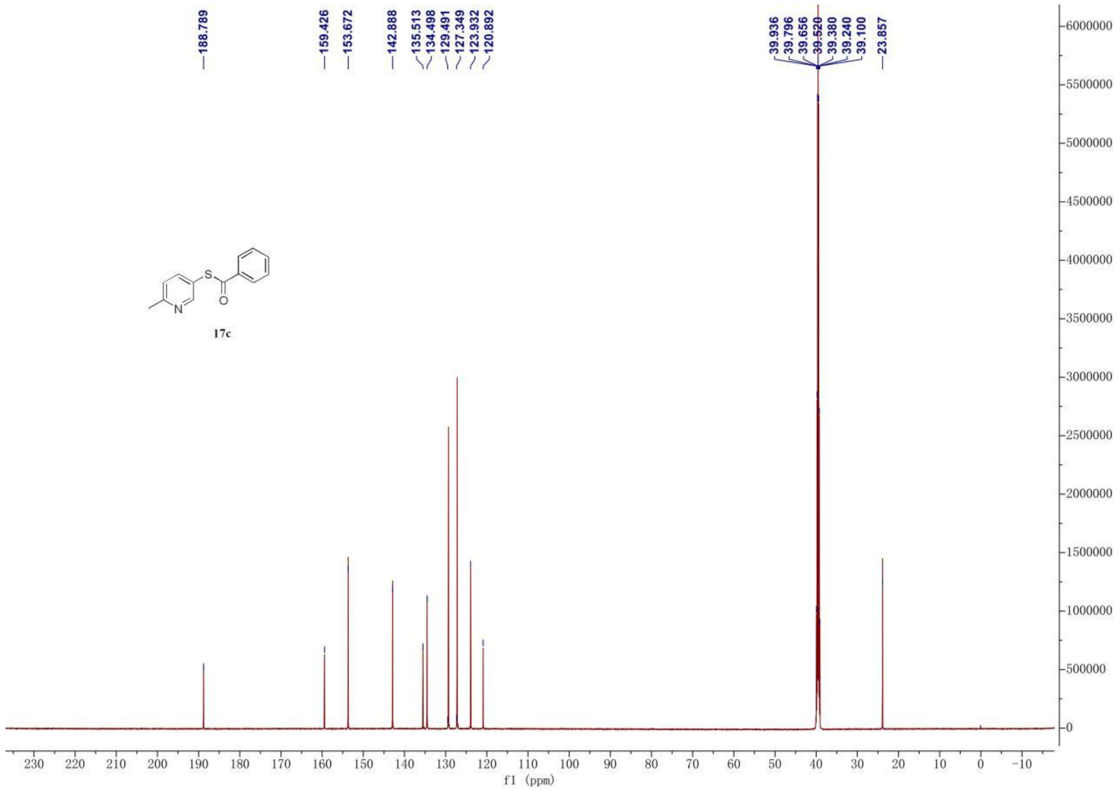

^{13}C NMR (151 MHz, DMSO-d_6) spectrum of **17c**

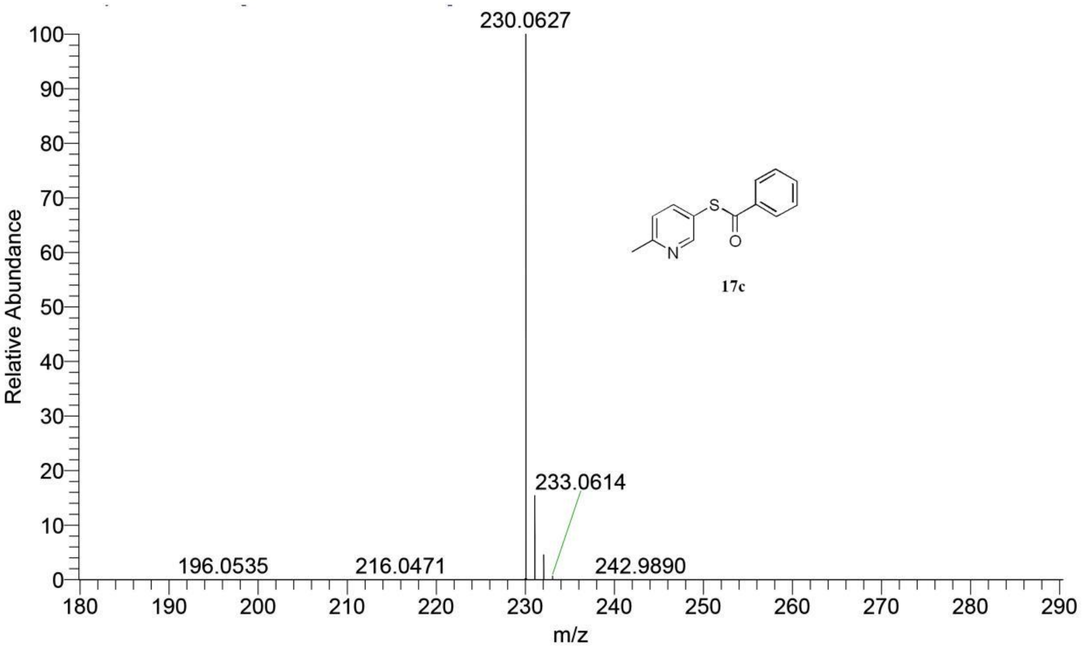

HRMS spectrum of **17c**

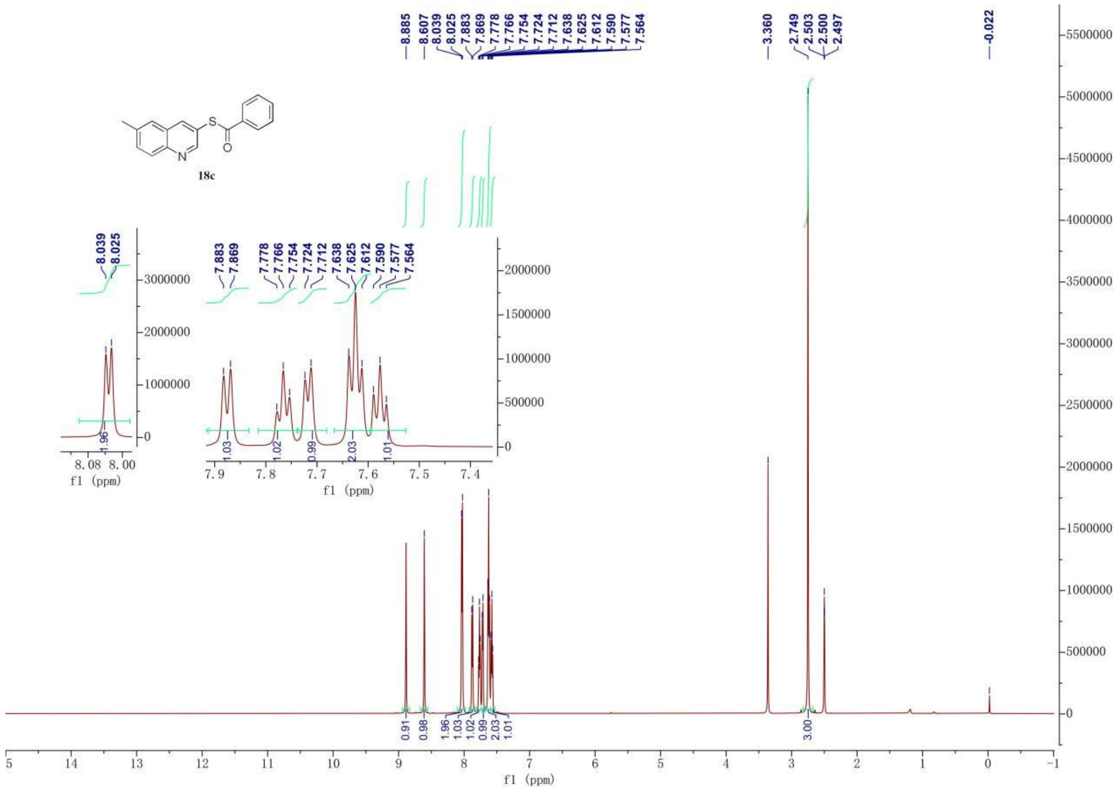

^{1}H NMR (600 MHz, DMSO-d_6) spectrum of **18c**

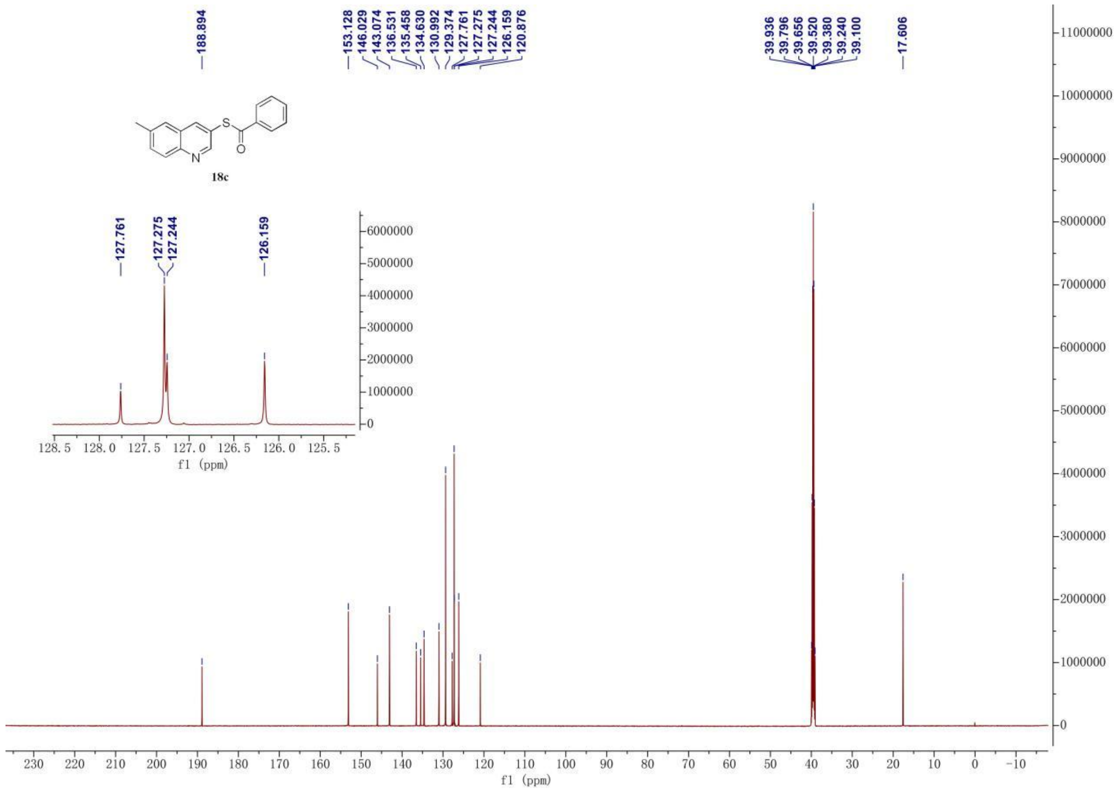

^{13}C NMR (151 MHz, DMSO-d_6) spectrum of **18c**

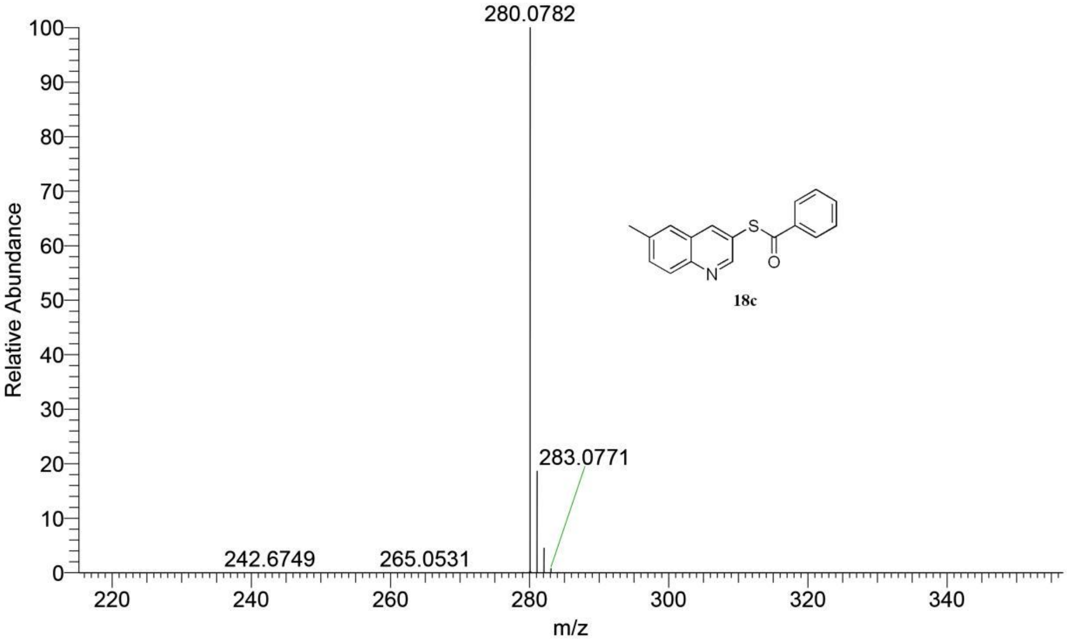

HRMS spectrum of **18c**

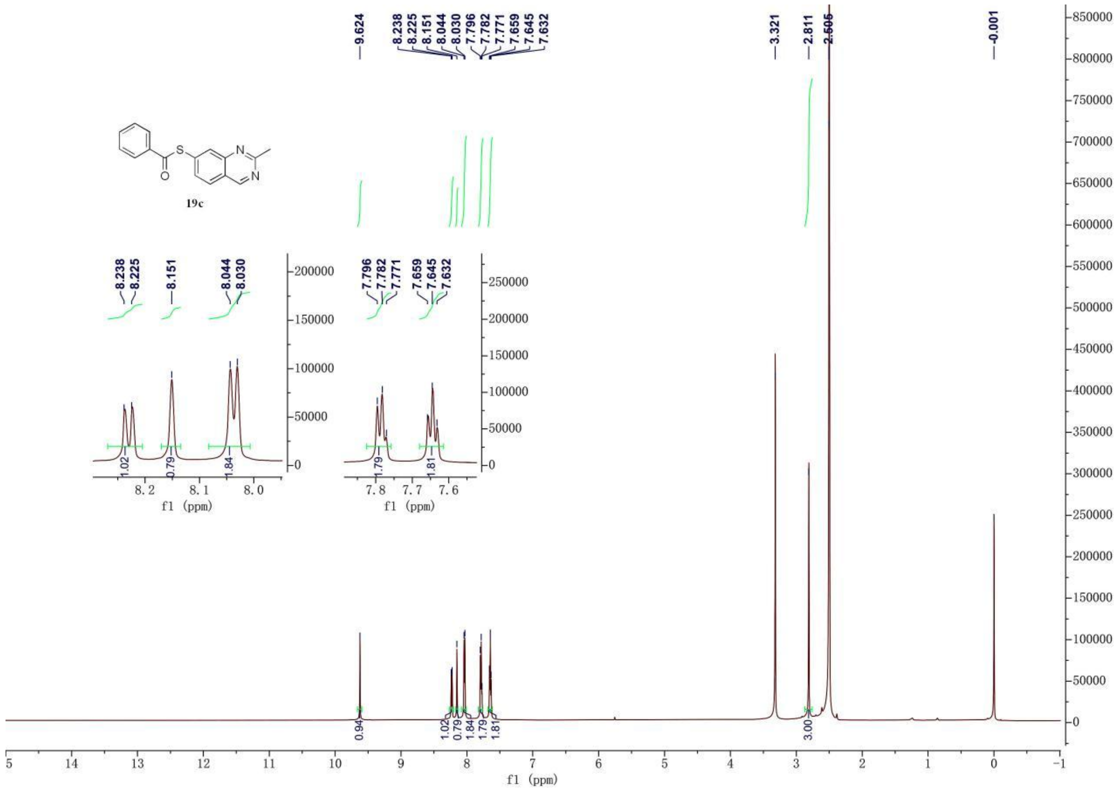

^{1}H NMR (600 MHz, DMSO-d_6) spectrum of **19c**

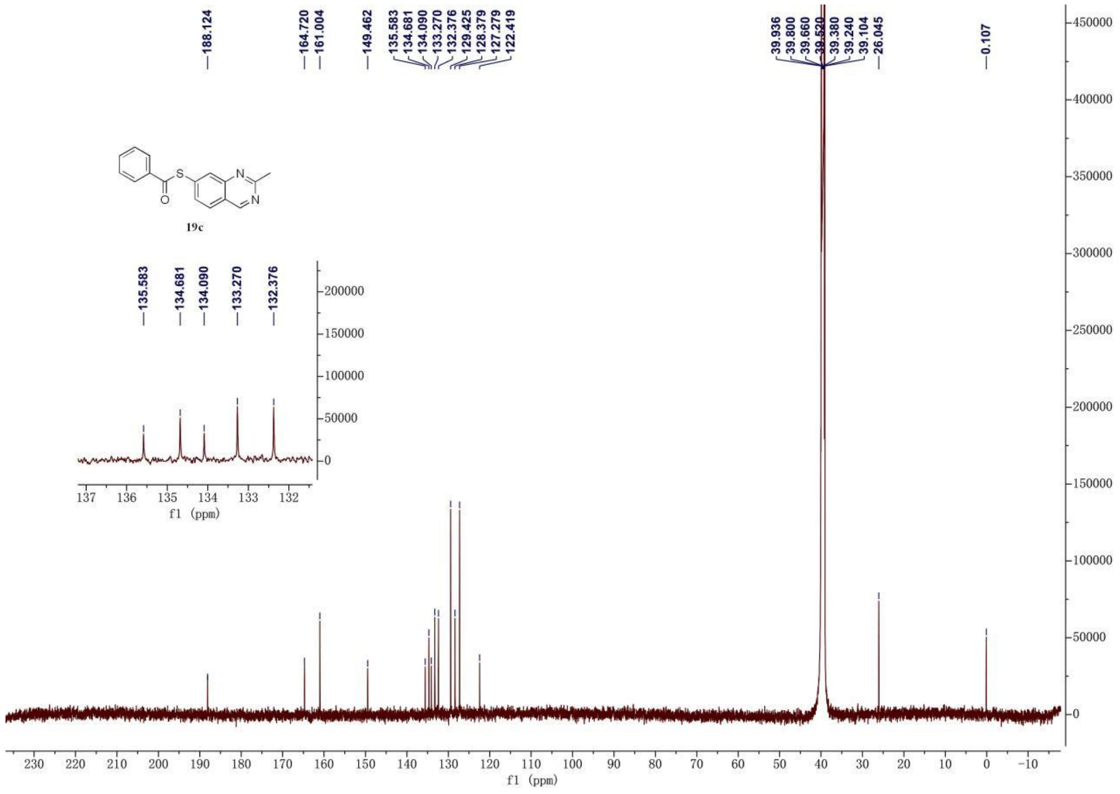

^{13}C NMR (151 MHz, DMSO-d_6) spectrum of **19c**

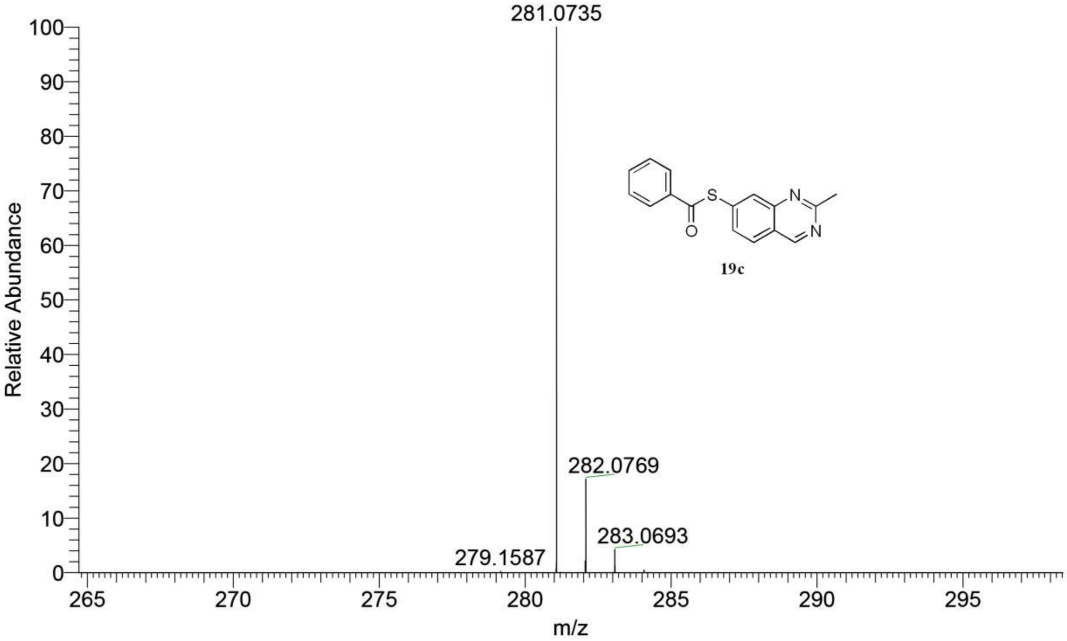

HRMS spectrum of **19c**

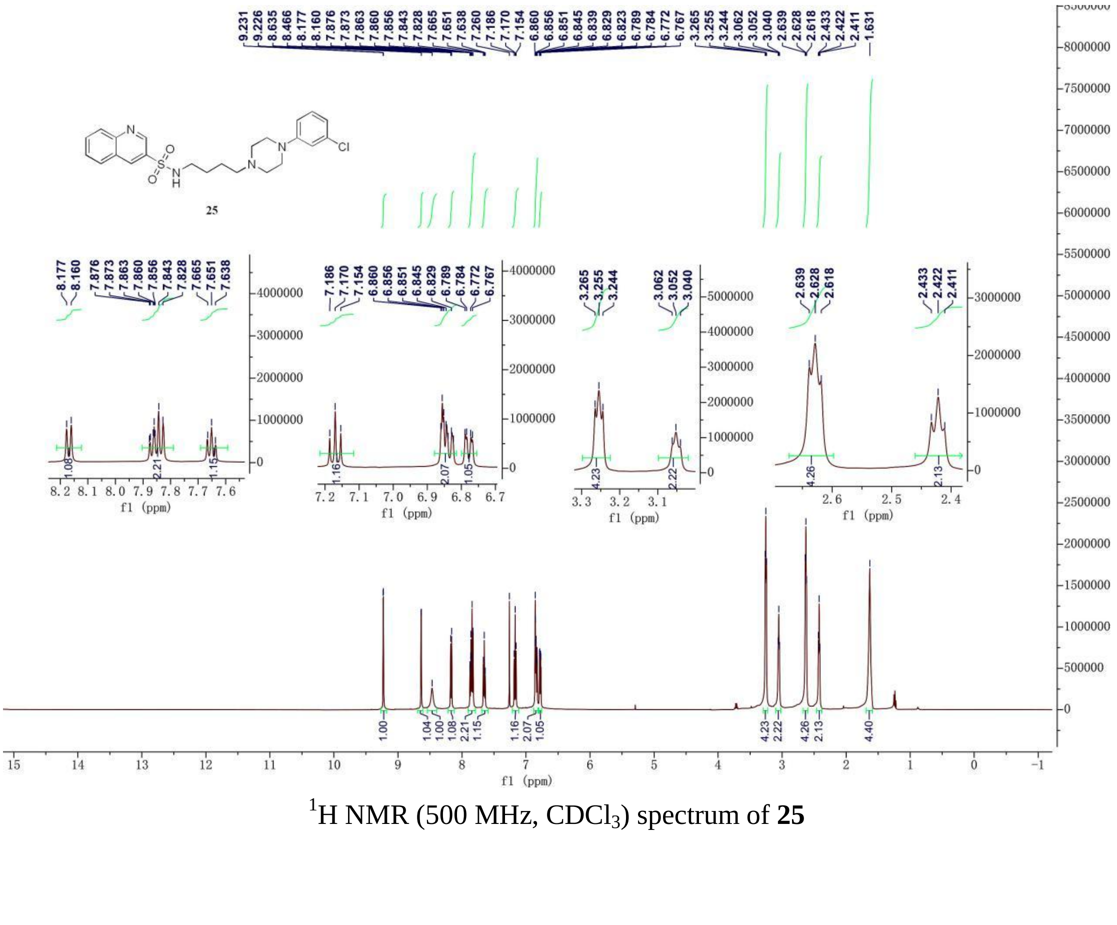

^{1}H NMR (500 MHz, $CDCl_3$) spectrum of **25**

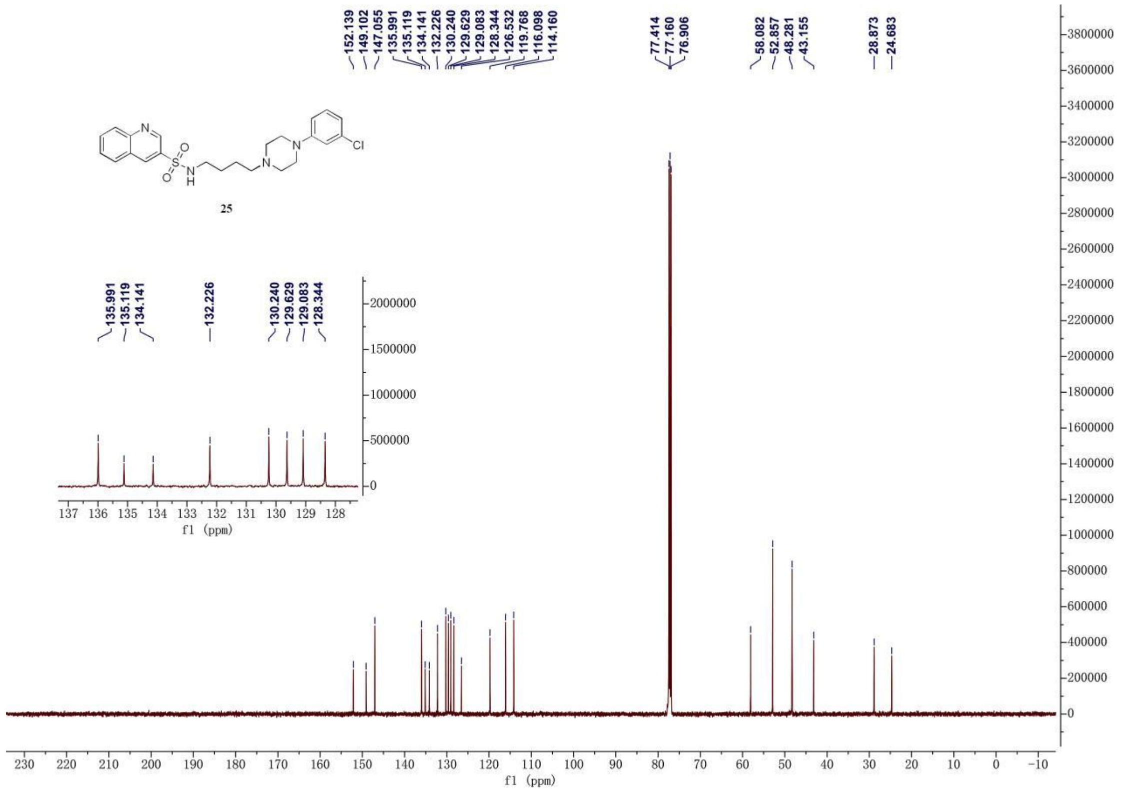

^{13}C NMR (126 MHz, $CDCl_3$) spectrum of **25**

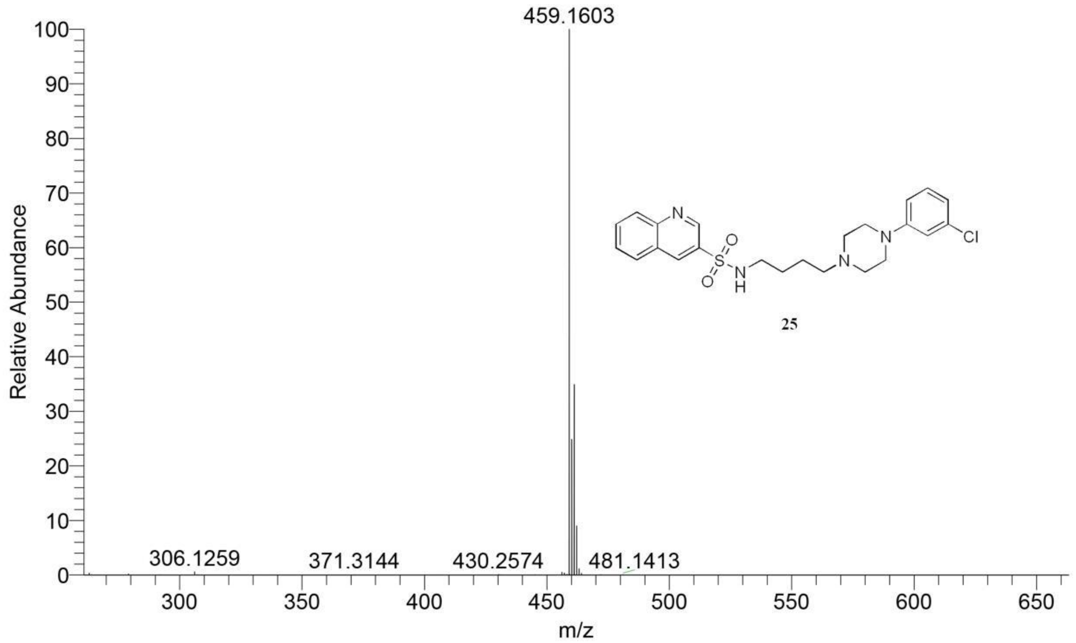

HRMS spectrum of **25**

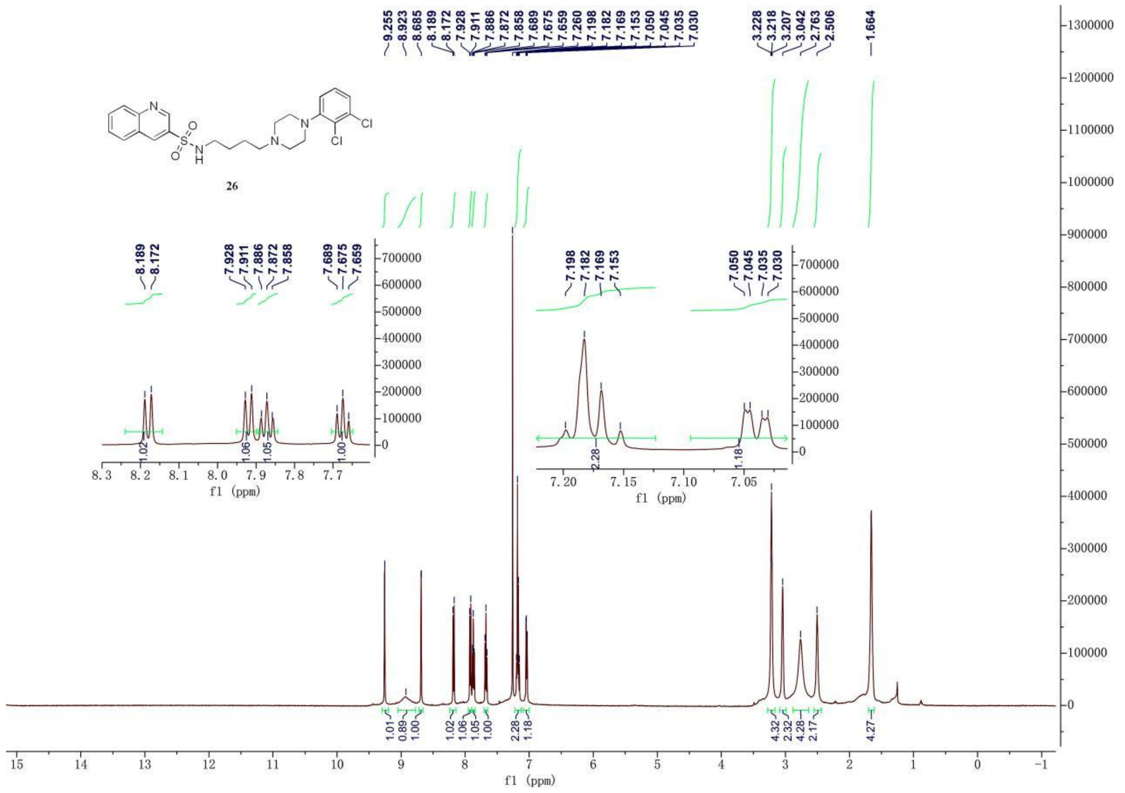

^{1}H NMR (500 MHz, $CDCl_3$) spectrum of **26**

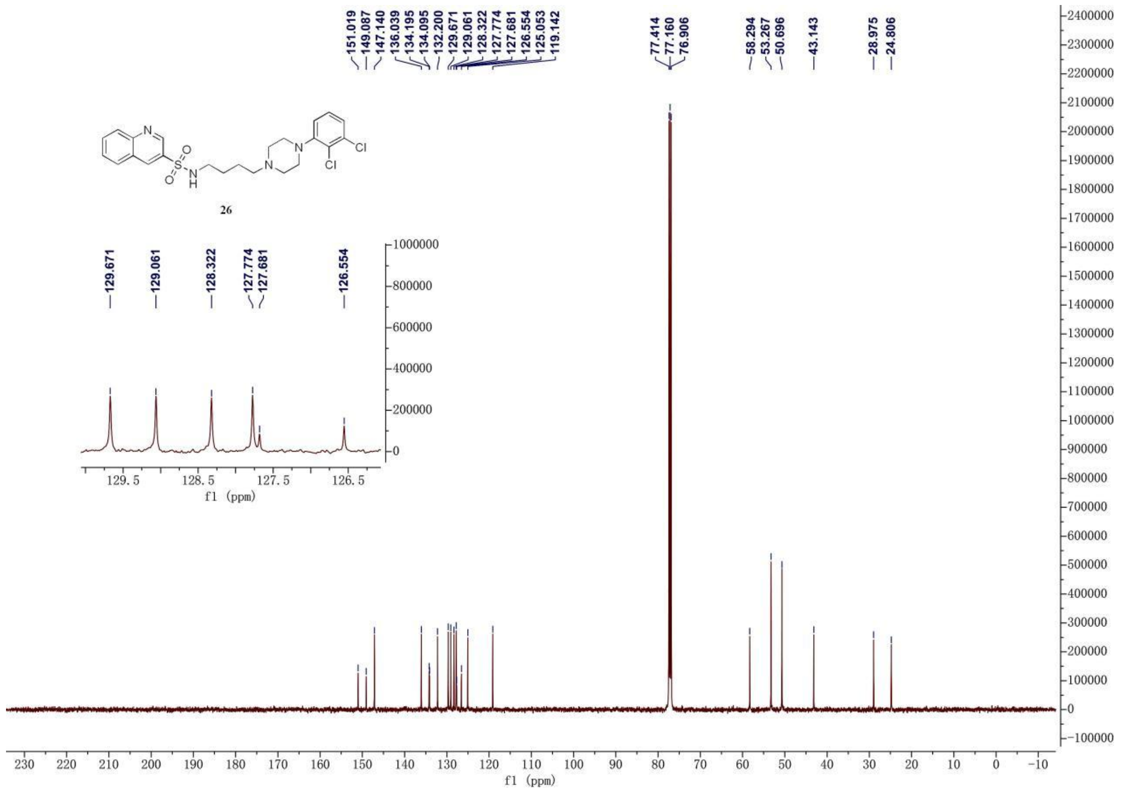

^{13}C NMR (126 MHz, $CDCl_3$) spectrum of **26**

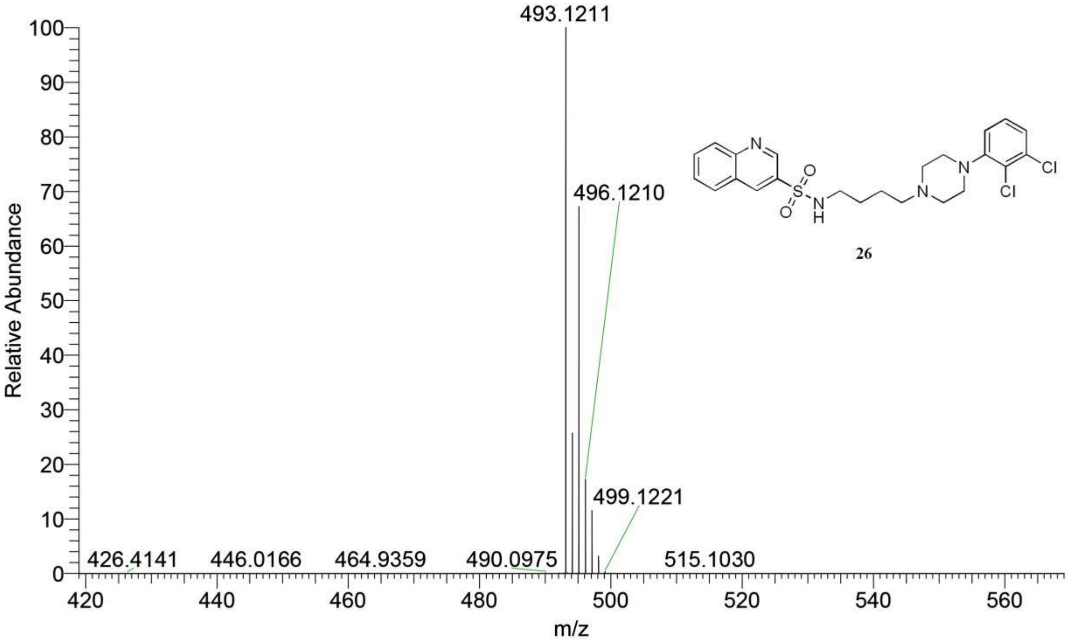

HRMS spectrum of **26**
